# Enhanced Antibacterial Activity of *Ent*-Labdane Derivatives of Salvic Acid (7α-Hydroxy-8(17)-*ent-*Labden-15-Oic Acid): Effect of Lipophilicity and the Hydrogen Bonding Role in Bacterial Membrane Interaction

**DOI:** 10.3390/molecules22071039

**Published:** 2017-06-23

**Authors:** Javier Echeverría, Alejandro Urzúa, Loreto Sanhueza, Marcela Wilkens

**Affiliations:** 1 Facultad de Química y Biología, Universidad de Santiago de Chile, Casilla 40, Correo 33, Santiago 9170022, Chile; alejandro.urzua@usach.cl (A.U.); marcela.wilkens@usach.cl (M.W.); 2 Núcleo de Química y Bioquímica, Facultad de Ciencias, Universidad Mayor, Santiago 8580745, Chile; loreto.sanhueza@umayor.cl

**Keywords:** salvic acid, antibacterial labdane-type diterpene, lipophilic acyl-labdanes derivatives, membrane disruption

## Abstract

In the present study, the antibacterial activity of several *ent*-labdane derivatives of salvic acid (7α-hydroxy-8(17)-*ent*-labden-15-oic acid) was evaluated in vitro against the Gram-negative bacterium *Escherichia coli* and the Gram-positive bacteria *Staphylococcus aureus* and *Bacillus cereus*. For all of the compounds, the antibacterial activity was expressed as the minimum inhibitory concentration (MIC) in liquid media and minimum inhibitory amount (MIA) in solid media. Structure activity relationships (SAR) were employed to correlate the effect of the calculated lipophilicity parameters (logP_ow_) on the inhibitory activity. Employing a phospholipidic bilayer (POPG) as a bacterial membrane model, *ent*-labdane-membrane interactions were simulated utilizing docking studies. The results indicate that (i) the presence of a carboxylic acid in the C-15 position, which acted as a hydrogen-bond donor (HBD), was essential for the antibacterial activity of the *ent*-labdanes; (ii) an increase in the length of the acylated chain at the C-7 position improved the antibacterial activity until an optimum length of five carbon atoms was reached; (iii) an increase in the length of the acylated chain by more than five carbon atoms resulted in a dramatic decrease in activity, which completely disappeared in acyl chains of more than nine carbon atoms; and (iv) the structural factors described above, including one HBD at C-15 and a hexanoyloxi moiety at C-7, had a good fit to a specific lipophilic range and antibacterial activity. The lipophilicity parameter has a predictive characteristic feature on the antibacterial activity of this class of compounds, to be considered in the design of new biologically active molecules.

## 1. Introduction

Many therapeutic small molecules used today as drugs have their origins in natural products (secondary metabolites), providing or inspiring the development of between 50% and 70% of all chemotherapeutic agents [1]. Antibiotics are one clear example and now there is particular concern over the ability of natural product investigations to yield new classes of antibacterial agents [2,3]. Although all early antibiotics were derived from natural sources, there have been no new clinically approved, product-based antibiotics discovered for over 30 years [4,5].

Within secondary metabolites of a terpenic nature, the labdane nucleus is a useful structure for the molecular exploration and development of new pharmaceutical compounds. The synthesis of labdane derivatives has received significant attention because labdane has proven to be important, as they possess pharmaceutical properties, including antibacterial (against different strains of both Gram-positive and Gram-negative bacteria) and antifungal activity, among other biological effects [6,7,8]. The success of this group of molecules has stimulated the search for new biologically active derivatives and research on the role and influence of chemical structure in biological activity. Progress in the use of structure-activity relationship (SAR) methods has shown the importance of the hydrophobicity or lipophilicity of biologically active molecules. Lipophilicity modifies the ability of bioactive molecules to penetrate through apolar parts of cell membranes. This property is usually characterized by the octanol-water partition coefficient (logP_ow_), which is essentially determined from distribution studies of a compound between an immiscible polar and non-polar solvent pair. This quantitative descriptor of lipophilicity is one of the key determinants of pharmacokinetic properties [9]. If the exact values for this parameter are known, the inhibitory activity of a related group of compounds can be predicted. In this context, the partition coefficient (logP), which is an index of lipophilicity, is an important physicochemical parameter in the development of antibacterial agents, because it is closely related to the permeation of these compounds through the structure of the bacterial lipid layer.

Docking studies provide valuable information on the interaction of active compounds with their biological targets [10,11]. Recently, docking studies suggested that diterpenes promote bacterial lysis due to their insertion into the lipophilic cell membrane and its consequent disruption [12]. According to these authors, the structural features related with the efficient antibacterial effects of these natural compounds include a lipophilic decalin ring system, which enables insertion into a lipophilic region, and a strategically positioned hydrogen-bond donor group (HBD; hydrophilic group), which promotes interactions with membrane phosphorylated groups. Moreover, in this study, a second HBD in the decalin ring system led to a reduction or suppression of activity.

**Figure 1:**
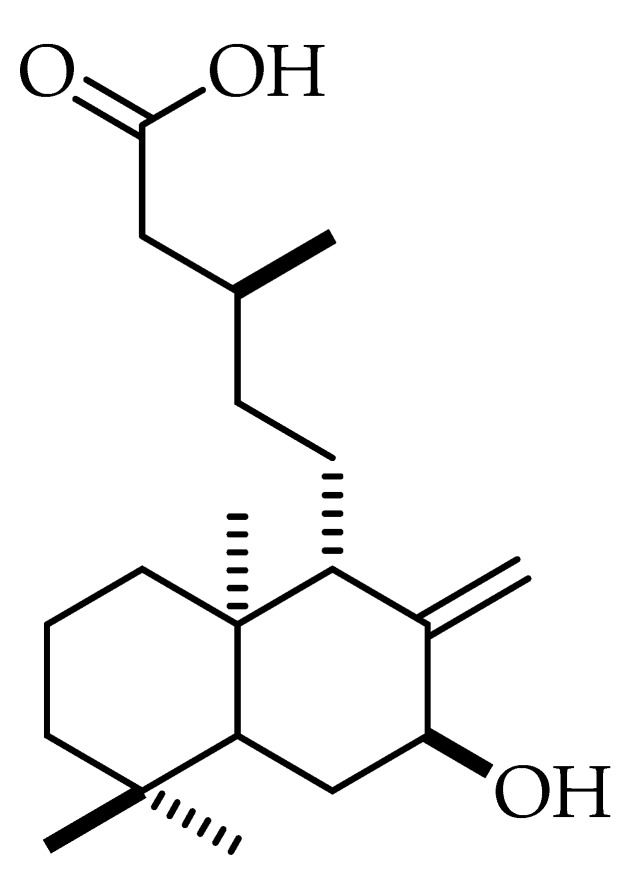
Structure of salvic acid.

In addition, and following our program in study of the SAR of antibacterial secondary metabolites of Chilean flora [13,14], lipophilic 7-*O*-esters with linear, branched, and unsaturated (C1 to C12) chains, 7-alkoxy,15-ester linear chain derivatives, 15-methylester, and 7,15-diol derivatives of salvic acid **1** (Figure 1) were synthesized; we tested the antimicrobial activity of these compounds against two Gram-positive (*Staphylococcus aureus* and *Bacillus cereus*) and one Gram-negative (*Escherichia coli*) bacteria to systematically evaluate the role of the HBD and the effect of lipophilicity on antibacterial activity. Additionally, we examined the interaction of 7-*O*-acyl salvic acid derivatives with the lipid bilayer (POPG), as an artificial model bacterial membrane. The model allowed us to correlate the antibacterial activity of the 7-*O*-acyl derivatives with their logP_ow_.

## 2. Results and Discussion

**Scheme 1:**
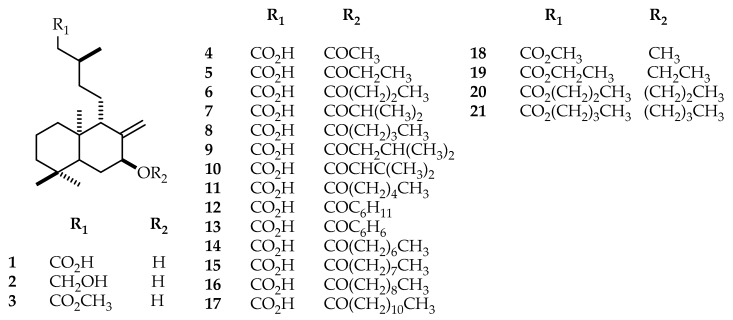
Structure of the hemisynthetic derivatives of salvic acid.

The hemisynthesis of salvic acid derivatives (Scheme 1) involved reduction, methylation, acylation, and alkoxylation-esterification reactions. These reactions were performed to increase the lipophilicity and to assess the significance of hydrogen-bond donor (HBD) groups in antibacterial activity. For this purpose, a set of salvic acid derivatives was obtained, including 7,15-diol **2** and 15-methylester **3** analogs, fourteen 7-*O*-acyl derivatives **4**–**17**, and four 7-alkoxy,15-ester derivatives **18**–**21**. The absolute configuration of salvic acid, which has been recently assigned, along with information obtained via X-ray diffraction and vibrational circular dichroism analysis [15], was used to verify the stereochemistry of the hemisynthetic derivatives.

### 2.1. Antibacterial Activity of the Salvic Acid Derivatives

The obtained MIC and MIA values are shown in Table 1. The MIC values in liquid media were not significantly different compared to the MIA values in solid media, although the latter showed greater differentiation in detailed SAR analysis.

The results showed a selective inhibitory effect that *ent*-labdane type diterpenes display in general on the growth of Gram-positive bacteria. None of tested compounds were active against Gram-negative bacteria *E. coli* (MIC up to 10 μg/mL and MIA up to 10 μg).

Compounds **8**–**12** and **14** in liquid media and **5** in solid media, showed the strongest antibacterial activity against *S. aureus*. Compounds **6**, **8**–**11**, and **12** in liquid media and **11** in solid media were the most active against *B. cereus*.

The results revealed that compounds with one HBD group, such as methylester **3** and some acyl derivatives, such as 7-*O*-hexanoyl salvic acid **11**, were the most actives ones. MIC and MIA values of several acyl derivatives of salvic acid were similar to those of reference antibiotics, suggesting further studies for possible clinical use.

### 2.2. Lipophilicity and Structure-Activity Relationship of Antibacterial of Salvic Acid and Its Derivatives

Lipophilicity, which is well correlated with the bioactivity of a chemical compound, is an important molecular descriptor, and the lipophilic behavior of a compound plays a significant role in their mechanism of biological activity. All of the compounds synthesized herein showed greater lipophilicity values than salvic acid **1**. SAR analysis of acyl derivatives, particularly between pairs **6**–**7** and **8**–**9**, suggested that the ramification of the hydrocarbon chain of the acyl group of C-7 caused a significant decrease in the MIC values in both Gram-positive bacteria. The observed decrease in MIC values was closely related to an increase in lipophilicity values. In contrast to the results described above, SAR analysis of acylated derivatives **9**, **10**, **12**, and **13** showed that the presence of insaturations in the hydrocarbon chain of the acyl group at C-7 caused an increase in MIC values, which in turn was related to the observed reduction in lipophilicity values.

The lipophilicity values (logP_ow_) of hemisynthetic derivatives (Table 1) of compounds with acyl groups, including compounds **4**–**17**, exhibited a gradual increase as the chain length of the acyl group increased; these derivatives showed greater lipophilicity compared to salvic acid **1**. The antibacterial activity of this series increased throughout the series up to a maximum, corresponding to compound **11**, then decreased sharply to compound **15**, disappearing completely in acyl derivatives with greater chain lengths, such as compounds **16**–**17**. These results show that the linear relationship between lipophilicity and activity did not continue ad infinitum. Therefore, a complete regression analysis was applied, including linear, quadratic, and cubic relationships. The data show that the fitting equations improved when higher-order (second- or third-order) polynomials were employed [16].

Careful observation of the results depicted in Table 1 shows that the reduction of the CO_2_H group of **1** to yield diol **2** increased the lipophilicity, but resulted in a loss of antibacterial activity (MIC up to 10 μg/mL and MIA up to 10 μg), suggesting that the presence of a carboxylic acid group strategically located at C-15 is important for the antimicrobial activity of the labdanes. Esterification of **1** to yield methylester **3** increased both the lipophilicity and antibacterial activity. These results show that the presence of only one HBD significantly improved the antimicrobial effect of the labdane-type diterpenes compared to the labdanes with two HBD groups (salvic acid **1** and diol **2**).

The importance of only one HBD was proven in the series of 7-alkoxy,15-ester derivatives, in which blocking the two HBD groups of salvic acid **1** was performed through alkoxylation and esterification reactions to yield compounds **18**–**21**; this synthetic modification caused an absolute inhibition of antibacterial activity, despite the large increase in logP_ow_ values. However, the presence of the anchor HBD group allowed these compounds to interact with the surface of the phospholipid bilayer, and because the lipophilicity of the decalin moiety is enhanced by the presence of the C-7 acyl group, the penetration of microorganisms through the lipid layer or disruption of the components of the bacterial membrane easily occurs [17]. These results were in agreement with our recent research, in which we demonstrated that a lipophilic decalin ring system with a strategically positioned HBD (hydrophilic group) is important for the antimicrobial activity of diterpenes. Moreover, in the same study, a second HBD introduced in the decalin ring system led to a reduction in or suppression of the activity. We argued that there were basically two mechanisms for the reduced antibacterial activity of diterpenoids containing two HBD groups: (i) the presence of two HBDs decreases the lipophilicity of the hydrophobic moiety, hindering its interaction with the bacterial membrane; (ii) intramolecular HBD group interactions compete with intermolecular hydrogen bonds between each HBD and the cell membrane [12]. This model has been successfully applied to other labdanes [18,19] and other types of diterpenes, including pimaranes and abietanes [20,21,22,23,24,25] and pseudopterane diterpene [26]. Further membrane-diterpene interaction studies have been performed, specifically in labdanes, e.g., sclareol, labd-7,13-dien-15-ol, and labd-13-ene-8α,15-diol, [27,28], (8*R*,13*S*)-labdane-8,15-diol and (8*R*,13*R*)-labdane-8,15-diol [29], myriadenolide [30], as well as antibacterial diterpenes that interact with bacterial membrane models, e.g., (+)-totarol, [31,32] and abietic acid [33,34], demonstrating that lipophilicity plays an important role in addition to structural factors. Also, among natural pentacyclic triterpenes, many reports have been published concerning the interaction between the lipid membrane and lupane triterpenoids [35,36,37,38,39,40]. The common functionality in these compounds is the hydroxyl group at C3. In addition, ursolic acid has an additional carboxylic acid function at C17. Literature reports have shown that the acid moiety at C17 and the esterification of C3-OH are essential for pentacyclic triterpenes with enhanced pharmacological activities. Based on these observations, the C3-OH can be derivatized with lipophilic ester chains.

### 2.3. Molecular Modeling: Docking Studies of Labdane-Membrane Interactions

**Figure 2:**
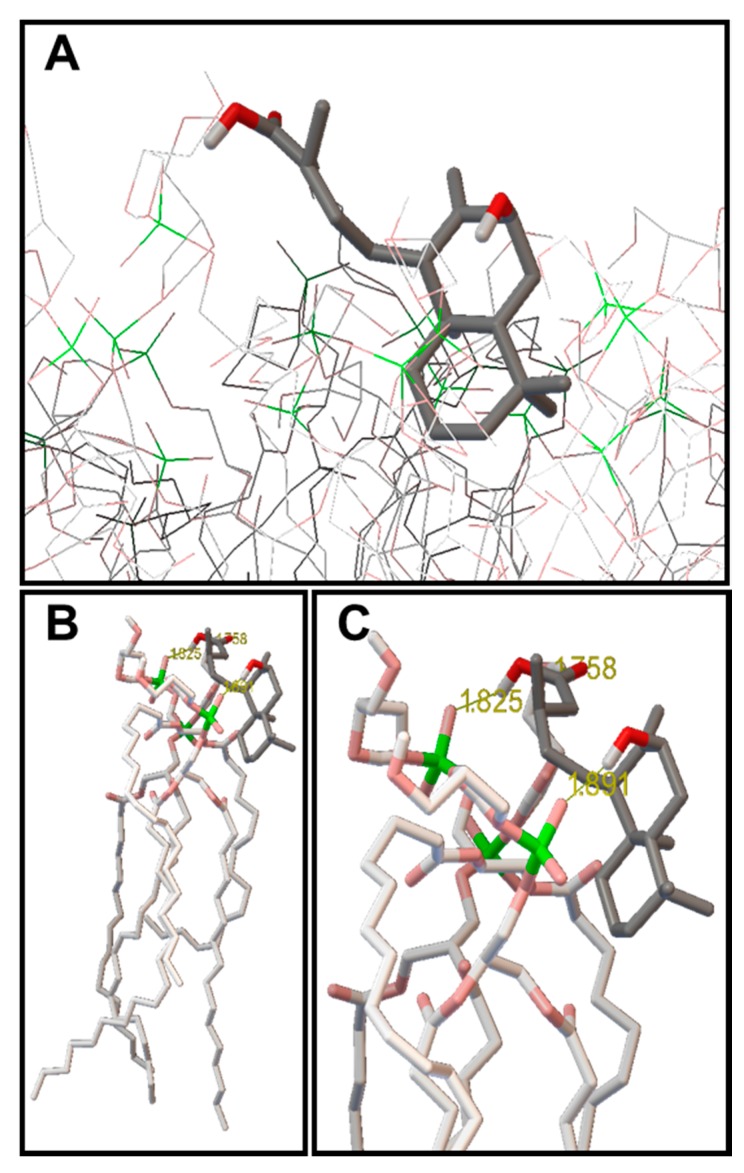
(**A**) Cross-sectional view of the spatial conformation that adopts compound **1** by interacting with the phospholipid bilayer (POPG); (**B**,**C**) Interactions by the formation of hydrogen bonds between compound **1** and POPG.

A molecular docking study was performed using optimized structures of the labdane derivatives and a phospholipidic bilayer (POPG) as a bacterial membrane model. This study was performed to corroborate our hypothesis and to examine the structural and functional factors that influence the mode of action in antibacterial diterpenes. The observed bond distances between the HBD groups of the diterpenes and the HBA (hydrogen bond acceptors) atoms of the phospholipids in POPG membranes, as shown in Figure 2, indicate that this type of interaction may be a significant contributor to the association, integration, and interaction of labdanes in the bacterial membrane model.

Interactions between salvic acid **1** and the POPG model membrane, as shown in Figure 2, account for the strong bonding, which was stabilized by the formation of three hydrogen bridge bonds. The first interaction occurred between the hydrogen atom of the carboxylic acid group and the O13 oxygen atom of the phosphatidylglycerol phosphate groups, with a C15(O)OH---O=P bond distance of 1.825 Å. In the same way, the hydrogen atom of the hydroxyl group formed a hydrogen bond with the O13 atom of another phospholipid, with a C7-OH---O=P bond distance of 1.891 Å. Finally, we observed that the oxygen atom of the hydroxyl group of the carboxylic acid acted as an acceptor, and the hydrogen bonds formed by the H16 hydrogen atom and glycerol hydroxyl of another phospholipid exhibited a C7-HO---HO bond distance of 1.758 Å. The conformation adopted by salvic acid on the surface of the bacterial membrane model was primarily located in the polar area of the bilayer POPG, with a small portion of the decalin skeleton introduced into the hydrophobic zone of the bilayer. These interactions explain the low antibacterial activity of salvic acid **1**, against both Gram-positive bacteria and the correlation with their lipophilicity value.

**Figure 3:**
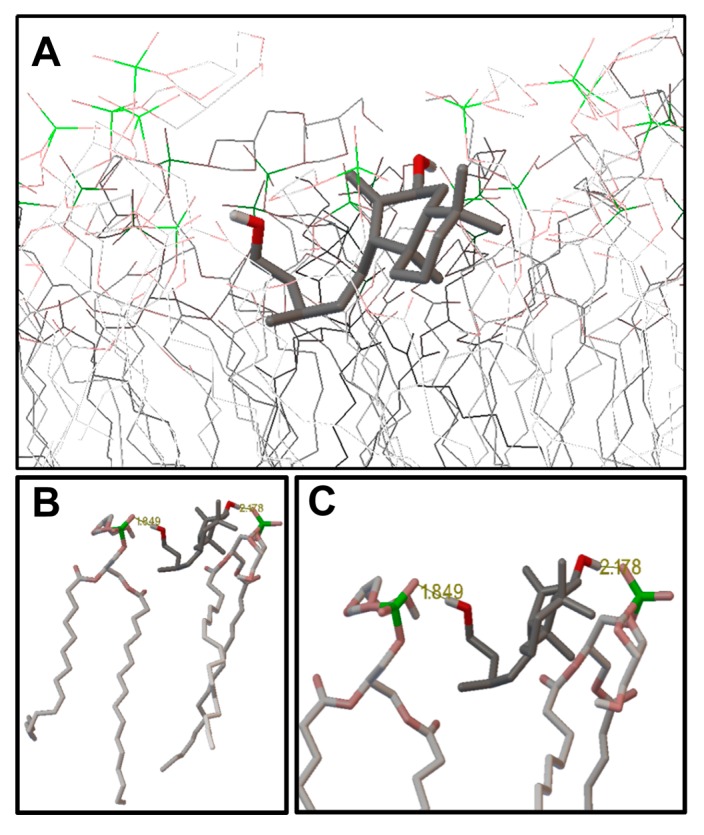
(**A**) Cross-sectional view of the spatial conformation that compound **2** adopts by interacting with the phospholipid bilayer POPG; (**B**,**C**) Interaction by the formation of hydrogen bonds between compound **2** and the nearest two phospholipid units of the POPG bilayer.

In the same way, the interpretation given to the interaction of salvic acid **1** with the POPG model membrane can be applied to the other derivatives with two HBD groups, including diol derivative **2**, as shown in Figure 3. In this case, the derivative interacted with the POPG bilayer through the formation of hydrogen bonds with the two hydrophilic groups, C7-OH and C15-OH. Both groups were formed through an interaction with the oxygen atoms of the phosphate groups of the two phospholipids, the first bond having a C7-OH---O=P distance of 1.849 Å and the second bond with a C15(O)-OH---O=P bond distance of 2.178 Å.

In contrast, the methyl ester derivative **3** showed an increase in both lipophilicity and antibacterial activity, reflected in the conformation that it adopts when interacting with the phospholipid bilayer, showing that the decalin is more introduced in the lipophilic zone of the membrane.

**Figure 4:**
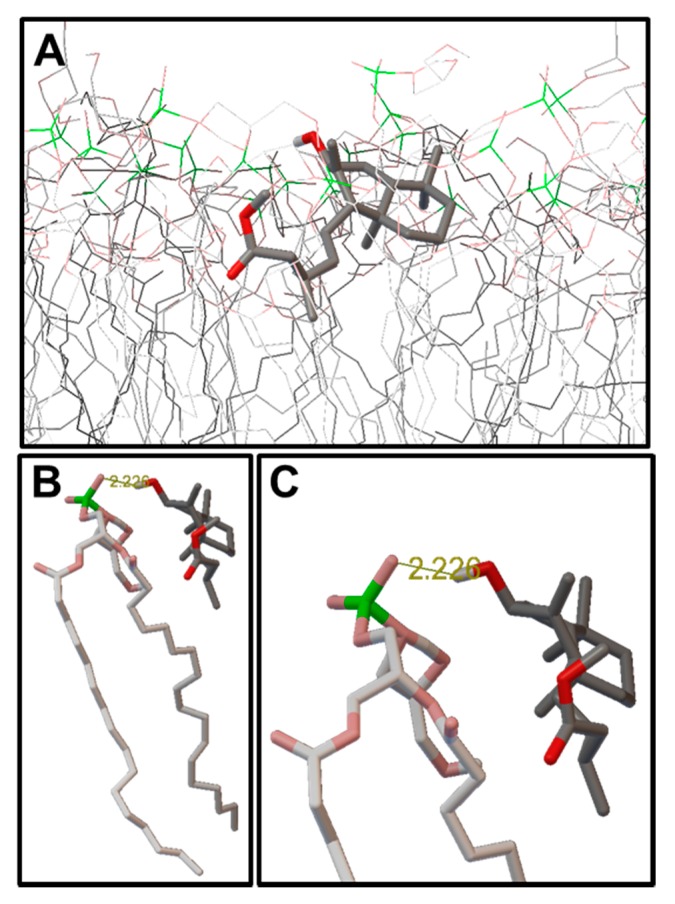
(**A**) Cross-sectional view of the spatial conformation that compound **3** adopts by interacting with the phospholipid bilayer POPG; (**B**,**C**) Interaction by the formation of hydrogen bonds between the compound **3** and one POPG.

This finding suggests a greater effect on membrane disruption, due to the formation of a hydrogen bond bridge between the C7-OH---O=P group, with a distance of 2.226 Å, favoring the anchoring of this compound to the phospholipid matrix (Figure 4).

**Figure 5:**
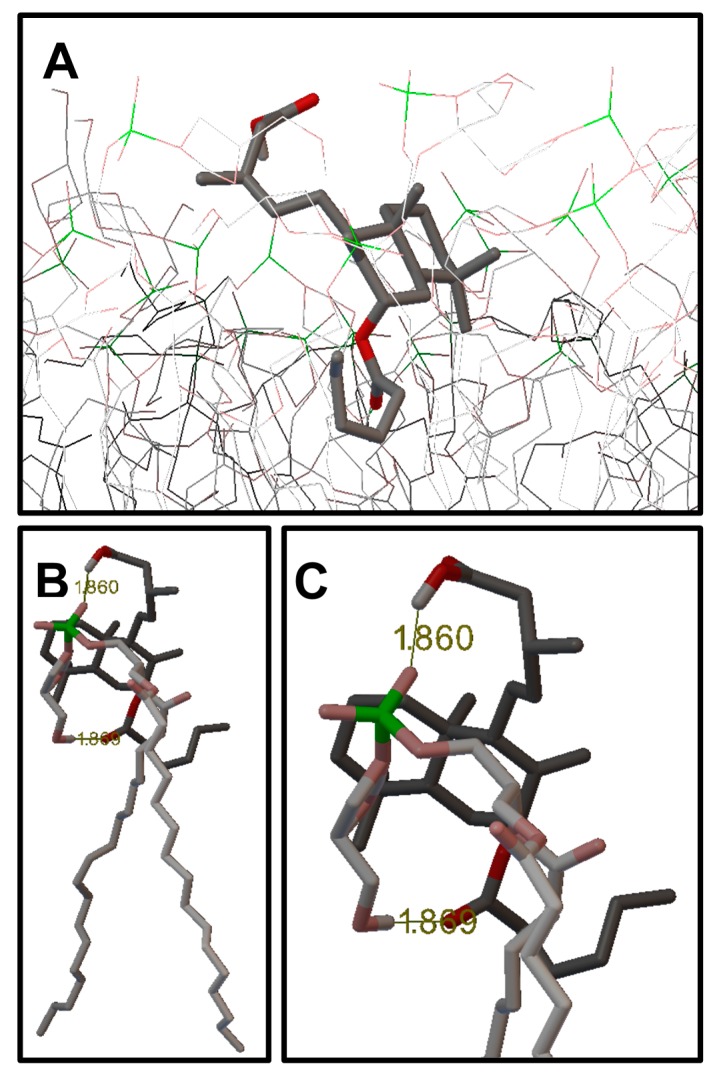
(**A**) Cross-sectional view of the spatial conformation that compound **11** adopts by interacting with the phospholipid bilayer POPG; (**B**,**C**) Interaction by the formation of hydrogen bonds between the compound **11** and one of the phospholipid bilayer POPGs.

In the conformations of the 7-*O*-acyl labdane series, a sequential increase was observed in the penetration of the acyl chain into the POPG membrane, as shown in Figure 5. This degree of penetration was correlated with the maximum antibacterial effect of 7-*O*-hexanoyl derivative **11**, the most active compound against both Gram-positive bacteria. These conformations were stabilized by the formation of two hydrogen bridge bonds, the first of which was stabilized by a donation between C15(O)-OH---O=P, with a distance of 1.860 Å, allowing the compound to act as an anchor in the phospholipid matrix. The second oxygen atom of the carbonyl group of the acyl moiety acted as a hydrogen bond acceptor to one of the hydroxyl groups of the phospholipid glycerolphosphate, with a C7–OCOR_2_---HO bond distance of 1.869 Å. For example, in derivative **17**, although a hydrogen bond had formed in C15-(O)OH---O=P, the interaction was very weak because the bond distance was 3.223 Å. Subsequent increases in the alkyl chain of the acyl group over the six carbon atoms hindered the penetration of labdane in the membrane, likely due to electrostatic and hydrophobic repulsions between the hydrocarbon chains of the acyl group and the polar groups of the phospholipids; in addition to steric effects associated with the increased volume and length of the chain. As a result, a drastic decrease in activity was observed until the antibacterial effect was completely inhibited, as exemplified in 7-*O*-lauriloxi derivative **17**, due to its interaction with POPG (Figure 4).

**Figure 6:**
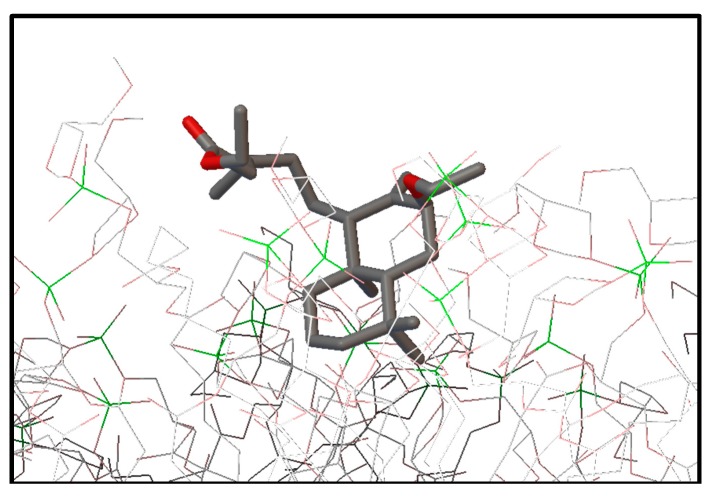
Cross-sectional view of the spatial conformation which compound **19** adopts by interacting with the phospholipid bilayer POPG, where the formation of hydrogen bonds is not observed.

Finally, we evaluated the conformations of the 7-alkoxy, 15-ester derivatives, which have significant structural characteristics, such as an absence of HBD groups, resulting in a low affinity for the POPG bilayer (Figure 6). Although we did not observe the formation of any hydrogen bridge bonds, the hydrophobic fraction was positioned toward the interior of the bilayer but was not firmly bonded, indicating that this conformation was transient, which explains the absence of activity.

The observations described above were based on experimental studies of labdane incorporation in the liposomes of phosphatidylcholine and demonstrate that labdanes possessing a single HBD were better incorporated into liposomes than labdanes with two HBDs [28,41]. The presence of two HBDs reduced its incorporation into the membrane, while the acetylation of these HBD groups resulted in increased incorporation.

For optimal interaction with amphipathic membranes, the presence of a polar substituent is required, preferably a carboxylic acid group, which acts as a HBD, in association with a lipophilic skeleton, primarily located in the labdane side chain. The presence of a second HBD group in the molecule reduces the lipophilicity of the hydrophobic moiety, which hinders interactions with the hydrophobic region of the bacterial membrane. Interestingly, the observed reduction in activity due to the presence of a hydrophilic group in a labdane-type diterpene is not limited to the compounds previously studied [12].

## 3. Materials and Methods

### 3.1. General

NMR spectra were obtained on a Bruker DPX 400 spectrometer (400 MHz for ^1^H and 100 MHz for ^13^C). Samples were dissolved in CDCl_3_, and the spectra were calibrated using TMS signals. The chemical shifts are given in ppm. The carbon atoms in the alkyl chains of the acyloxy groups were numbered by labelling the carbon atom of the carbonyl group as number one and subsequently increasing towards the methyl terminus of the acyl chain. All ^1^H-NMR spectra were been added in Supplementary Materials.

### 3.2. Plant Material

Aerial portions of resinous *Eupatorium salvia* Colla were collected from Cuesta Lo Prado (Región Metropolitana, Chile, 31°28′ S, 71°27′ W) at an altitude of 785 m above the average sea level during the flowering season in March of 2008. Voucher specimens were deposited in the Herbarium of the National Museum of Natural History, Santiago, Chile (SGO 108833).

### 3.3. Extraction and Isolation of Salvic Acid *(**1**)*

Aerial portions of *E. salvia* (1.8 kg) were extracted by dipping fresh plant material into 5 L of CH_2_Cl_2_ at room temperature for 30 s. This procedure was repeated twice to ensure the total and selective extraction of epicuticular components. The combined CH_2_Cl_2_ extracts were evaporated, and the residue (133 g) was fractionated by column chromatography on silica gel (CC) using pentane/CH_2_Cl_2_ and CH_2_Cl_2_/CH_3_OH step gradients. The fractions were eluted with CH_2_Cl_2_/CH_3_OH (99:1) and spontaneously crystallized to yield 40 g of salvic acid **1**, which was identified by direct comparison with an authentic sample [12,42].

### 3.4. Reduction of Salvic Acid: Diol *(**2**)*

To a solution of 300 mg (0.93 mmol) of salvic acid **1** in dry ethyl ether, a suspension of lithium aluminum hydride (200 mg, 5.3 mmol) in anhydrous ethyl ether (20 mL) was carefully added dropwise under a nitrogen atmosphere. The reaction mixture was refluxed for 1 h and washed successively with a solution of 5% hydrochloric acid and distilled water. The resulting organic phase was dried over anhydrous sodium sulfate, and the solvent was removed on a rotary evaporator to yield 282.8 mg (0.92 mmol, 99%) of a white crystalline compound. The partial spectral data of **2** were in agreement with published results [43]; therefore, the full NMR assignment is presented.

*7β,15-dihydroxy-ent-lab-8(17)-ene* (**2**) (yield 99%, 282.8 mg): ^1^H-NMR (CDCl_3_, 400 MHz) δ 5.04 (1H, t, *J* = 1.3 Hz, H-17β), 4.63 (1H, t, *J* = 1.6 Hz, H-17α), 4.38 (1H, d, *J* = 2.9 Hz, H-7), 3.68 (1H, m, H-15α), 3.66 (1H, m, H-15β), 2.05 (1H, m, H-9), 1.78 (1H, d, *J* = 10.2 Hz, H-1α), 1.66 (1H, dd, *J* = 7.5; 2,1 Hz, H-14α), 1.59 (1H, s, H-5), 1.58 (1H, s, H-11α), 1.57 (2H, d, *J* = 3.0 Hz, H-6), 1.56 (1H, d, *J* = 3.0 Hz, H-2α), 1.54 (1H, m, H-13), 1.50 (1H, m, H-2β), 1.44 (1H, dd, *J* = 7.5; 3.8 Hz), 1.42 (1H, m, H-3α), 1.37 (1H, m, H-14β), 1.26 (1H, dd, *J* = 9.0; 3.8 Hz, H-11β), 1.24 (1H, dd, *J* = 12.8; 5,2 Hz, H-3β), 1.09 (1H, td, *J* = 12.3; 4.1 Hz, H-1β), 0.98 (1H, dd, *J* = 11.3; 6.3 Hz, H-12β), 0.91 (3H, d, *J* = 6.5 Hz, H-16), 0.88 (3H, s, H-18), 0.80 (3H, s, H-19), 0.65 (3H, s, H-20). ^13^C-NMR (CDCl_3_, 101 MHz) δ: 149.82 (C=CH_2_, C-8), 109.72 (C=CH_2_, C-17), 74.16 (CH, C-7), 61.23 (CH_2_, C-15), 51.40 (CH, C-9), 47.65 (CH, C-5), 42.07 (CH_2_, C-3), 39.88 (C, C-10), 39.59 (CH_2_, C-14), 38.78 (CH_2_, C-1), 35.91 (CH_2_, C-12), 33.30 (CH_3_, C-18), 33.09 (C, C-4), 30.88 (CH_2_, C-6), 30.20 (CH, C-13), 21.51 (CH_3_, C-19), 20.43 (CH_2_, C-11), 19.83 (CH_3_, C-16), 19.34 (CH_2_, C-2), 13.39 (CH_3_, C-20).

### 3.5. Methylation of Salvic Acid: Methylsalvate *(**3**)*

A solution of 200 mg (0.62 mmol) of salvic acid **1** in dry ethyl ether (15 mL) was treated with a solution of diazomethane in ethyl ether, which was obtained from the treatment of *N*-methyl-*N*-nitroso-*p*-toluene sulfonamide (Diazald) with a concentrated solution of potassium hydroxide. The aqueous phase was discarded, and the organic phase was successively dried with potassium hydroxide beads in an Erlenmeyer flask. The solvent was removed using a rotary evaporator, yielding 208.7 mg (0.62 mmol, 100%) of methylsalvate **3**.

### 3.6. Acylation of Salvic Acid: 7-O-Acyl Derivatives *(**4**–**17**)*

To a solution of 200 mg (0.62 mmol) of salvic acid **1** in dichloromethane (20 mL), the appropriate choice of acyl chloride or acid anhydride (0.7 mL) was added, followed by 4-*N*, *N*-dimethylaminopyridine (DMAP) (80 mg, 0.7 mmol). The reaction mixture was stirred at room temperature for 24 h and was successively washed with a 5% solution of hydrochloric acid, a 6% solution of sodium bicarbonate, and distilled water. The resulting organic phase was dried over anhydrous sodium sulfate, and the solvent was removed using a rotary evaporator to give the corresponding acyl derivative. The reaction product was purified by CC with a CH_2_Cl_2_–CH_3_OH gradient to yield the respective derivatives. To identify C and H atoms, the acyl group of the C-7 position was labelled using the prime (′) symbol and was used as a starting point for enumeration. Thus, the atoms were numbered in ascending order, starting with 1′ at the C=O.

*7-O-Acetyl salvic acid* (**4**) (Yield: 90%, 203 mg); ^1^H-NMR (CDCl_3_, 400 MHz) δ: 5.42 (1H, s, H-7), 5.15 (1H, s, H-17α), 4.72 (1H, s, H-17β), 2.38 (1H, dd, *J* = 15.0; 5.5 Hz, H-14α), 2.12 (1H, dd, *J* = 15.1; 8.2 Hz, H-14β), 2.05 (3H, s, H-2′), 1.89 (1H, s, H-13), 1.85 (1H, d, *J* = 2.8 Hz, H-6), 1.76 (1H, d, *J* = 12.6 Hz, H-1α), 1.60 (1H, m, H-6), 1.54 (2H, d, *J* = 13.6 Hz, H-2), 1.48 (1H, s, H-5), 1.44 (1H, d, *J* = 4.5 Hz, H-12 α), 1.42 (1H, d, *J* = 12.7 Hz, H-3), 1.26 (2H, m, H-11), 1.21 (1H, m, H-3), 1.10 (1H, dd, *J* = 12.6; 4.0 Hz, H-1β), 1.02 (1H, d, *J* = 4.8 Hz, H-12β), 0.98 (3H, d, *J* = 6.6 Hz, H-13), 0.83 (3H, s, H-18), 0.79 (3H, s, H-19), 0.67 (3H, s, H-20). ^13^C-NMR (CDCl_3_, 101 MHz) δ: 178.52 (C-15), 170.41 (C-1′), 145.20 (C-8), 112.18 (C-17), 76.43 (C-7), 52.32 (C-9), 48.71 (C-5), 42.05 (C-3), 41.08 (C-14), 39.54 (C-10), 38.80 (C-1), 35.21 (C-12), 33.27 (C-18), 33.07 (C-4), 30.80 (C-13), 28.93 (C-6), 21.57 (C-19), 21.39 (C-2), 20.55 (C-11), 19.96 (C-16), 19.34 (C-2), 13.55 (C-20).

*7-O-Propionyl salvic acid* (**5**) (Yield: 97%, 229 mg); ^1^H-NMR (CDCl_3_, 400 MHz) δ: 5.41 (1H, s, H-7), 5.16 (1H, s, H-17α), 4.72 (1H, s, H-17β), 2.38 (1H, dd, *J* = 15.0; 5.5 Hz, H-14α), 2.32 (2H, q, *J* = 7.6 Hz, H-2′), 2.12 (1H, dd, *J* = 15.0; 8.4 Hz, H-14β), 1.94 (1H, d, *J* = 10.5 Hz, H-9), 1.93 (2H, m, H-6), 1.89 (1H, m, H-13), 1.76 (1H, d, *J* = 12.3 Hz, H-1α), 1.54 (2H, dd, *J* = 13.5; 2.8 Hz, H-2), 1.49 (1H, s, H-5), 1.40 (1H, s, H-3), 1.26 (2H, m, H-11), 1.22 (1H, dd, *J* = 13.4; 4.3 Hz, H-3β), 1.14 (3H, t, *J* = 7.6 Hz, H-3′), 1.06 (1H, dd, *J* = 12.7; 4.6 Hz, H-1), 1.00 (2H, m, H-12), 0.97 (3H, d, *J* = 6.7 Hz, H-16), 0.82 (3H, s, H-18), 0.79 (3H, s, H-19), 0.68 (3H, s, H-20). ^13^C-NMR (CDCl_3_, 101 MHz) δ: 179.46 (C-15), 173.76 (C-1′), 145.22 (C-8), 112.14 (C-17), 76.19 (C-7), 52.34 (C-9), 48.76 (C-5), 42.06 (C-3), 41.18 (C-14), 39.53 (C-10), 38.81 (C-1), 35.22 (C-12), 33.28 (C-18), 33.08 (C-4), 30.79 (C-13), 30.76 (C-6), 28.19 (C-2′), 21.39 (C-19), 20.53 (C-11), 19.98 (C-16), 19.40 (C-3′), 19.33 (C-2), 13.54 (C-20).

*7-O-Butyryl salvic acid* (**6**) (Yield: 88%, 214 mg); ^1^H-NMR (CDCl_3_, 400 MHz) δ: 5.42 (1H, s, H-7), 5.16 (1H, s, H-17α), 4.72 (1H, s, H-17β), 2.38 (1H, dd, *J* = 15.0; 4.6 Hz, H-14α), 2.28 (2H, t, *J* = 7.4 Hz, H-2′), 2.11 (1H, dd, *J* = 15.0; 8.5 Hz, H-14β), 1.94 (1H, d, *J* = 10.2 Hz, H-9), 1.92 (2H, m, H-6), 1.88 (1H, s, H-13), 1.76 (1H, d, *J* = 12.6 Hz, H-1α), 1.65 (2H, qd, *J* = 7.4; 1.3 Hz, H-3′), 1.55 (2H, d, *J* = 13.9 Hz, H-2), 1.49 (1H, s, H-5), 1.45 (1H, m, H-12α), 1.40 (1H, s, H-3α), 1.25 (2H, m, H-11), 1.21 (1H, td, *J* = 13.2; 3.3 Hz, H-3β), 1.09 (1H, td, *J* = 10.8; 3.1 Hz, H-1β), 1.01 (1H, d, *J* = 6.5 Hz, H-12β), 0.97 (3H, d, *J* = 6.7 Hz, H-16), 0.94 (3H, t, *J* = 7.4 Hz, H-4′), 0.83 (3H, s, H-18), 0.79 (3H, s, H-19), 0.67 (3H, s, H-20). ^13^C-NMR (CDCl_3_, 101 MHz) δ: 179.01 (C-15), 172.92 (C-1′), 145.24 (C-8), 112.07 (C-17), 76.04 (C-7), 52.39 (C-9), 48.78 (C-5), 42.07 (C-3), 41.14 (C-14), 39.52 (C-10), 38.81 (C-1), 36.84 (C-2′), 35.22 (C-12), 33.26 (C-18), 33.08 (C-4), 30.77 (C-13), 29.00 (C-6), 21.39 (C-19), 20.52 (C-11), 19.94 (C-16), 19.33 (C-2), 18.70 (C-3′), 13.70 (C-4′), 13.56 (C-20).

*7-O-Isobutyryl salvic* acid (**7**); (Yield: 86%, 210 mg); ^1^H-NMR (CDCl_3_, 400 MHz) δ: 5.39 (1H, s, H-7), 5.15 (1H, s, H-17α), 4.71 (1H, s, H-17β), 2.54 (1H, sept, *J* = 7.0 Hz, H-2′), 2.38 (1H, dd, *J* = 15.0; 5.4 Hz, H-14α), 2.11 (1H, dd, *J* = 15.0; 8.6 Hz, H-14β), 1.95 (1H, t, *J* = 9.4 Hz, H-9), 1.92 (2H, m), 1.87 (1H, m, H-13), 1.76 (1H, d, *J* = 12.6 Hz, H-1α), 1.53 (2H, m, H-2), 1.49 (1H, m, H-5), 1,44 (1H, m, H-12α), 1.40 (1H, m, H-3α), 1.26 (2H, m, H-11), 1.21 (1H, d, *J* = 7.0 Hz, H-3β), 1.16 (3H, d, *J* = 6.4 Hz, H-3′), 1.15 (3H, d, *J* = 6.6 Hz, H-4′), 1.07 (1H, dd, *J* = 12.5; 4.0 Hz, H-1β), 1.00 (1H, m, H-12β), 0.97 (3H, d, *J* = 6.6 Hz, H-16), 0.82 (3H, s, H-18), 0.79 (3H, s, H-19), 0.67 (3H, s, H-20). ^13^C-NMR (CDCl_3_, 101 MHz) δ: 178.91 (C-15), 176.25 (C-1′), 145.10 (C-8), 112.01 (C-17), 75.79 (C-7), 52.40 (C-9), 48.81 (C-5), 42.02 (C-3), 41.05 (C-14), 39.46 (C-10), 38.77 (C-1), 35.13 (C-12), 34.31 (C-2′), 33.23 (C-18), 33.02 (C-4), 30.71 (C-13), 28.85 (C-6), 21.34 (C-19), 20.38 (C-11), 19.90 (C-16), 19.27 (C-2), 19.13 (C-3′), 18.94 (C-4′), 13.50, (C-20).

*7-O-Valeroyl salvic acid* (**8**) (Yield: 84%, 212 mg); ^1^H-NMR (CDCl_3_, 400 MHz) δ: 5.42 (1H, s, H-7), 5.15 (1H, s, H-17α), 4.71 (1H, s, H-17β), 2.38 (1H, dd, *J* =14.9; 5.4 Hz, H-14), 2.30 (2H, t, *J* = 7.4 Hz, H-2′), 2.11 (1H, dd, *J* = 15.0; 8.5 Hz, H-14β), 1.94 (1H, d, *J* = 11.8 Hz, H-9), 1.92 (2H, *J* = 14.1 Hz, H-6), 1.87 (1H, s, H-13), 1.76 (1H, t, *J* = 12.6 Hz, H-1α), 1.59 (2H, dd, *J* = 15.0; 7.5 Hz, H-3′), 1.52 (2H, d, *J* = 13.6 Hz, H-2), 1.48 (1H, s, H-5), 1.44 (1H, m, H-12α), 1.40 (1H, m, H-3β), 1.35 (2H, dd, *J* = 15.0, 7.5 Hz, H-4′), 1.25 (2H, m, H-11), 1.22 (1H, dd, *J* = 13.6; 4.5 Hz, H-3β), 1.08 (1H, d, *J* = 12.6, 3.7 Hz, H-1β), 1.02 (1H, m, H-12β), 0.98 (3H, d, *J* = 6.6 Hz, H-16), 0.91 (3H, t, *J* = 7.3 Hz, H-5′), 0.82 (3H, s, H-18), 0.79 (3H, s, H-19), 0.67 (3H, s, H-20). ^13^C-NMR (CDCl_3_, 101 MHz) δ: 178.45 (C-15), 173.11 (C-1′), 145.20 (C-8), 112.08 (C-17), 76.01 (C-7), 52.37 (C-9), 48.75 (C-5), 42.05 (C-3), 41.02 (C-14), 39.50 (C-10), 38.79 (C-1), 35.21 (C-12), 34.64 (C-2′), 33.24 (C-18), 33.07 (C-4), 30.79 (C-13), 28.96 (C-6), 27.32 (C-3′), 22.23 (C-4′), 21.39 (C-19), 20.52 (C-11), 19.94 (C-16), 19.32 (C-2), 13.79 (C-5′), 13.55 (C-20).

*7-O-Isovaleroyl salvic acid* (**9**) (Yield: 77%, 196 mg); ^1^H-NMR (CDCl_3_, 400 MHz) δ: 5.44 (1H, s, H-7), 5.16 (1H, s, H-17α), 4.71 (1H, s, H-17β), 2.38 (1H, dd, *J* = 15.0; 5.5 Hz, H-14α), 2.18 (2H, d, *J* = 7.1 Hz, H-2′, 2.10 (1H, m, H-3′), 1.94 (1H, d, *J* = 9.4 Hz, H-9), 1.92 (2H, m, H-6), 1.87 (1H, s, H-13), 1.76 (1H, d, *J* = 12.6 Hz, H-1α), 1.54 (2H, d, *J* = 13.4 Hz, H-2), 1.49 (1H, m, H-5), 1.44 (1H, d, *J* = 6.7 Hz, H-12α), 1.40 (1H, s, H-3 α), 1.26 (2H, m, H-11), 1.21 (1H, td, *J* = 13.0, 4.1 Hz), 1.08 (1H, td, *J* = 12.6; 3.6 Hz, H-1β), 1.04 (1H, d, *J* = 5.8 Hz, H-12β), 0.97 (3H, d, *J* = 6.7 Hz, H-16), 0.95 (3H, d, *J* = 6.7 Hz, H-4′), 0.95 (3H, d, *J* = 6.7 Hz, H-5′), 0.82 (3H, s, H-18), 0.79 (3H, s, H-19), 0.67 (3H, s, H-20). ^13^C-NMR (CDCl_3_, 101 MHz) δ: 179.19 (C-15), 172.42 (C1′), 145.25 (C-8), 112.11 (C-17), 75.95 (C-7), 52.41 (C-9), 48.80 (C-5), 44.05 (C-2′), 42.08 (C-3), 41.18 (C-14), 39.50 (C-10), 38.80 (C-1), 35.24 (C-12), 33.24 (C-18), 33.11 (C-4), 30.78 (C-13), 29.05 (C-6), 25.92 (C-3′), 22.44 (C-5′), 22.36 (C-4′), 21.40 (C-19), 20.54 (C-11), 19.93 (C-16), 19.33 (C-2), 13.57 (C-20).

*7-O-Senecioyl salvic acid* (**10**) (Yield: 79%, 196 mg); ^1^H-NMR (CDCl_3_, 400 MHz) δ: 5.69 (1H, s, H-2′), 5.43 (1H, s, H-7), 5.17 (1H, s, H-17α), 4.71 (1H, s, H-17β), 2.39 (1H, dd, *J* = 15.0; 5.4 Hz, H-14α), 2.15 (3H, s, H-4′), 2.10 (1H, dd, *J* = 15.0; 8.7 Hz, H-14β), 1.96 (1H, d, *J* = 10.4 Hz, H-9), 1.92 (2H, m, H-6), 1.90 (1H, s, H-13), 1.89 (3H, s, H-5′), 1.76 (1H, d, *J* = 12.7 Hz, H-1α), 1.57 (2H, m, H-2), 1.50 (1H, s, H-5), 1.43 (1H, m, H-12α), 1.41 (1H, d, *J* = 12.5 Hz, H-3α), 1.26 (2H, m, H-11), 1.20 (1H, td, *J* = 13.2; 4,1 Hz, H-3β), 1.02 (1H, dd, *J* = 12.5; 5.0 Hz, H-1β), 1.01 (1H, d, *J* = 5.0 Hz, H-12β), 0.97 (3H, d, *J* = 6.6 Hz, H-16), 0.82 (3H, s, H-18), 0.79 (3H, s, H-19), 0.68 (3H, s, H-20). ^13^C-NMR (CDCl_3_, 100 MHz) δ: 178.84 (C-15), 166.06 (C-1′), 155.75 (C-3′), 145.45 (C-8), 116.81 (C-2′), 111.89 (C-17), 75.50 (C-7), 52.33 (C-9), 48.75 (C-5), 42.05 (C-3), 41.09 (C-14), 39.50 (C-10), 38.80 (C-1), 35.14 (C-12), 33.26 (C-18), 33.06 (C-4), 30.80 (C-13), 29.00 (C-6), 27.42 (C-5′), 21.42 (C-19), 20.51 (C-11), 20.26 (C-4′), 19.91 (C-16), 19.33 (C-2), 13.57 (C-20).

*7-O-Hexanoyl salvic acid* (**11**) (Yield 77%, 203 mg); ^1^H-NMR (CDCl_3_, 400 MHz) δ: 5.42 (1H, s, H-7), 5.16 (1H, s, H-17α), 4.72 (1H, s, H-17β), 2.30 (2H, t, *J* = 7.4 Hz, H-2′), 2.39 (1H, dd, *J* = 15.0; 5.3 Hz, H-14α), 2.11 (1H, dd, *J* = 15.0; 8.6 Hz, H-14β), 1.94 (1H, d, *J* = 11.4 Hz, H-9), 1.89 (1H, s, H-13), 1.76 (1H, d, *J* = 12.5 Hz, H-1α), 1.62 (2H, m, H-3′), 1.56 (1H, m, H-6), 1.54 (2H, m, H-2), 1.48 (1H, s, H-5), 1.44 (1H, m, H-12α), 1.40 (1H, s, H-2), 1.33 (2H, m, H-5′) 1.32 (2H, m, H-4′), 1.29 (2H, m, H-11), 1.21 (1H, td, *J* = 13.1; 3.9 Hz, H-3), 1.09 (1H, dd, *J* = 12.6; 3.9 Hz, H-1β), 1.03 (1H, dd, *J* = 12.4; 5.0 Hz, H-12β), 0.97 (3H, d, *J* = 6.6 Hz, H-16), 0.89 (3H, t, *J* = 6.7 Hz, H-6′), 0.82 (3H, s, H-18), 0.79 (3H, s, H-19), 0.67 (3H, s, H-20). ^13^C-NMR (CDCl_3_, 100 MHz) δ: 179.66 (C-15), 173.17 (C-1′), 145.15 (C-8), 112.09 (C-17), 76.07 (C-7), 52.33 (C-9), 48.73 (C-5), 42.04 (C-3), 41.21 (C-14), 39.48 (C-10), 38.77 (C-1), 35.19 (C-12), 34.86 (C-2′), 33.24 (C-18), 33.05 (C-4), 31.26 (C-4′), 30.76 (C-13), 28.94 (C-6), 24.90 (C-3′), 22.37 (C-5′), 21.38 (C-19), 20.50 (C-11), 19.94 (C-16), 19.31 (C-2), 13.93 (C-6′), 13.53 (C-20).

*7-O-Cyclohexanoyl salvic acid* (**12**) (Yield: 82%, 222 mg); ^1^H-NMR (CDCl_3_, 400 MHz) δ: 5.39 (1H, s, H-7), 5.15 (1H, s, H-17α), 4.71 (1H, s, H-17β), 2.38 (1H, dd, *J* = 10.6; 4.5 Hz, H-14α), 2.32 (1H, m, H-2′), 2.11 (1H, dd, *J* = 14.9; 8.5 Hz, H-14β), 1.95 (1H, s, H-9), 1.92 (2H, m, H-3′α), 1.87 (1H, m, H-6α), 1.87 (1H, s, H-13), 1.77 (1H, m, H-1α), 1.76 (2H, m, H-4′α), 1.65 (2H, m, H-5′α-β), 1.55 (1H, d, *J* = 3.0 Hz, H-6β), 1.54 (2H, dd, *J* = 14.0; 3.1 Hz, H-2), 1.48 (1H, s, H-5), 1.44 (2H, m, H-3′β), 1.42 (1H, m, H-12α), 1.40 (1H, td, *J* = 11.2; 3.4 Hz, H-3α), 1.28 (2H, m, H-11), 1.28 (2H, m, H-4′β), 1.21 (1H, td, *J* = 11.4; 2.8 Hz, H-3β), 1.10 (1H, dd, *J* = 12.7; 4.1 Hz, H-1β), 1.02 (1H, m, H-12β), 0.97 (3H, d, *J* = 6.6 Hz, H-16), 0.82 (3H, s, H-18), 0.79 (3H, s, H-19), 0.67 (3H, s, H-20). ^13^C-NMR (CDCl_3_, 100 MHz) δ: 178.88 (C-15), 175.29 (C-1′), 145.59 (C-8), 112.02 (C-17), 75.78 (C-7), 52.44 (C-9), 48.87 (C-5), 42.83 (C-2′), 42.09 (C-3), 41.11 (C-14), 39.52 (C-10), 38.83 (C-1), 35.18 (C-12), 33.31 (C-18), 33.08 (C-4), 30.76 (C-13), 29.20 (C-6), 28.81 (C-3′), 25.70 (C-4′), 25.35 (C-5′), 21.40 (C-19), 20.46 (C-11), 19.96 (C-16), 19.35 (C-2), 13.56 (C-20).

*7-O-Benzoyl salvic acid* (**13**) (Yield: 69%, 182 mg); ^1^H-NMR (CDCl_3_, 400 MHz) δ: 8.16 (2H, dd, *J* = 8.2; 1.4 Hz, H-3′), 7.69 (1H, tt, *J* = 7.6; 1.1 Hz, H-5′), 7.53 (2H, t, *J* = 7.9 Hz, H-4′), 5.53 (1H, t, *J* = 2.8 Hz, H-7), 5.13 (1H, s, H-17α), 4.69 (1H, s, H-17β), 2.28 (1H, dd, *J* = 13.8; 8.3 Hz, H-14α), 2.05 (1H, dd, *J* = 13.7; 6.2 Hz, H-14β), 1.98 (1H, s, H-9), 1.92 (1H, dd, *J* = 14.3; 7.0 Hz, H-13), 1.85 (1H, t, *J* = 2.6 Hz, H-6α), 1.79 (1H, m, H-1α), 1.58 (2H, td, *J* = 13.4; 3.2 Hz, H-2), 1.53 (1H, m, H-6β), 1.50 (1H, s, H-5), 1.44 (1H, m, H-12α), 1.43 (1H, d, *J* = 13.4 Hz, H-3α), 1.29 (2H, m, H-11), 1.22 (1H, m, H-3β), 1.08 (1H, td, *J* = 12.8; 3.7 Hz, H-1β), 1.00 (1H, m, H-12β), 0.92 (3H, d, *J* = 6.6 Hz, H-16), 0.83 (3H, s, H-18), 0.80 (3H, s, H-19), 0.68 (3H, s, H-20). ^13^C-NMR (CDCl_3_, 100 MHz) δ: 179.54 (C-15), 165.83 (C-1′), 145.17 (C-8), 132.77 (C-5′), 131.04 (C-2′), 129.45 (C-3′), 128.41 (C-4′), 112.45 (C-17), 76.36 (C-7), 52.66 (C-9), 49.28 (C-5), 42.09 (C-3), 41.19 (C-14), 39.59 (C-10), 38.88 (C-1), 35.17 (C-12), 33,36 (C-18), 33,07 (C-4), 30.71 (C-13), 29.16 (C-6), 21.40 (C-19), 20.58 (C-11), 20.00 (C-16), 19.37 (C-2), 13.56 (C-20).

*7-O-Octanoyl salvic acid* (**14**) (Yield: 79%, 221 mg); ^1^H-NMR (CDCl_3_, 400 MHz) δ: 5.41 (1H, s, H-7), 5.15 (1H, s, H-17α), 4.71 (1H, s, H-17β), 2.37 (1H, dd, *J* = 15.4; 5.5 Hz, H-14α), 2.34 (2H, t, *J* = 7.5 Hz, H-2′), 2.11 (1H, dd, *J* = 15.0; 8.5 Hz, H-14β), 1.95 (1H, s, H-9), 1.92 (1H, d, *J* = 1.9 Hz, H-13), 1.88 (1H, m, H-6α), 1.76 (1H, d, *J* = 12.8 Hz, H-1α), 1.63 (2H, dt, *J* = 14.8; 7.4 Hz, H-3′), 1.56 (1H, m, H-6β), 1.54 (1H, d, *J* = 13.9 Hz, H-2), 1.48 (1H, s, H-5), 1.44 (1H, m, H-3α), 1.40 (1H, m, H-12α), 1.31 (2H, m, H-4′), 1.30 (2H, m, H-5′), 1.28 (2H, m, H-7′), 1.28 (2H, m, H-11), 1.27 (2H, m, H-6′), 1.20 (1H, m, H-3β), 1.06 (1H, dd, *J* = 13.1; 4.5 Hz, H-1β), 1.02 (1H, dd, *J* = 12.1; 5.1 Hz, H-12β), 0.97 (3H, d, *J* = 6.6 Hz, H-16), 0.88 (3H, t, *J* = 6.0 Hz, H-8′), 0.82 (3H, s, H-18), 0.79 (3H, s, H-19), 0.67 (3H, s, H-20). ^13^C-NMR (CDCl_3_, 100 MHz) δ: 179.79 (C-15), 173.27 (C-1′), 145.39 (C-8), 112.22 (C-17), 76.23 (C-7), 52.55 (C-9), 48.93 (C-5), 42.22 (C-3), 41.28 (C-14), 39.66 (C-10), 38.96 (C-1), 35.38 (C-12), 35.07 (C-2′), 33.40 (C-18), 33.22 (C-4), 31.77 (C-6′), 30.94 (C-13), 29.26 (C-4′), 29.17 (C-5′), 29.04 (C-6), 25.38 (C-3′), 22.73 (C-7′), 21.53 (C-19), 20.68 (C-11), 20.11 (C-16), 19.48 (C-2), 14.18 (C-8′), 13.69 (C-20).

*7-O-Pelargonoyl salvic acid* (**15**) (Yield: 92%, 264 mg); ^1^H-NMR (CDCl_3_, 400 MHz) δ: 5.41 (1H, s, H-7), 5.15 (1H, s, H-17α), 4.71 (1H, s, H-17β), 2.39 (1H, dd, *J* = 15.1; 5.4 Hz, H-14α), 2.35 (2H, t, *J* = 7.5 Hz. H-2′), 2.12 (1H, dd, *J* = 15.0; 8.5 Hz, H-14β), 1.95 (1H, m, H-9), 1.92 (1H, s, H-13), 1.89 (1H, s, H-6α), 1.76 (1H, d, *J* = 12.5 Hz, H-1α), 1.63 (2H, m, H-3′), 1.56 (1H, m, H-6β), 1.54 (1H, d, *J* = 14.0 Hz, H-2), 1.48 (1H, s, H-5), 1.44 (1H, m, H-3α), 1.40 (1H, m, H-12α), 1.31 (2H, m, H-4′), 1.29 (2H, m, H-11), 1.29 (2H, m, H-5′), 1.29 (2H, m, H-8′), 1.27 (2H, m, H-6′), 1.27 (2H, m. H-7′), 1.21 (1H, td, *J* = 13.0; 3.9 Hz, H-3β), 1.06 (1H, dd, *J* = 13.5; 4.4 Hz, H-1β), 1.03 (1H, dd, *J* = 12.4; 5.1 Hz, H-12β), 0.96 (3H, d, *J* = 6.6 Hz, H-16), 0.88 (3H, t, *J* = 6.2 Hz, H-9′), 0.82 (3H, s, H-18), 0.79 (3H, s, H-19), 0.67 (3H, s, H-20). ^13^C-NMR (CDCl_3_, 100 MHz) δ: 178.34 (C-15), 173.09 (C-1′), 145.26 (C-8), 122.06 (C-17), 76.07 (C-7), 52.43 (C-9), 48.79 (C-5), 42.09 (C-3), 40.90 (C-14), 39.94 (C-2′), 39.52 (C-10), 38.82 (C-1), 35.24 (C-12), 33.27 (C-18), 33.09 (C-4), 31.85 (C-7′), 30.83 (C-13), 29.32 (C-4′), 29.18 (C-6′), 29.08 (C-5′), 28.98 (C-6), 25.24 (C-3′), 22.65 (C-8′), 21.40 (C-19), 20.55 (C-11), 19.96 (C-16), 19.34 (C-2), 14.08 (C-9′), 13.56 (C-20).

*7-O-Decanoyl salvic acid* (**16**) (Yield: 82%, 241 mg); ^1^H-NMR (CDCl_3_, 400 MHz) δ: 5.41 (1H, t, *J* = 2.5 Hz, H-7), 5.15 (1H, s, H-17α), 4.71 (1H, s, H-17β), 2.38 (1H, dd, *J* =15.0; 5.4 Hz, H-14α), 2.35 (1H, t, *J* = 7.6 Hz, H-2′α), 2.29 (1H, t, *J* = 7.4 Hz, H-2′β), 2.11 (1H, dd, *J* = 15.0; 8.5 Hz, H-14β), 1.95 (1H, m, H-9), 1.92 (1H, m, H-13), 1.88 (1H, m, H-6), 1.76 (1H, d, *J* = 12.6 Hz, H-1α), 1.63 (2H, m, H-3′), 1.54 (2H, dd, *J* = 13.7; 2.8 Hz, H-2), 1.48 (1H, s, H-5), 1.40 (1H, m, H-12α), 1.42 (1H, m, H-3α), 1.27–1.30 (12H, m, H-4′ to H-9′), 1.29 (2H, m, H-11), 1.21 (1H, td, *J* = 13.7; 4.6 Hz, H-3β), 1.06 (1H, dd, *J* = 13.1; 4.6 Hz, H-1β), 1.02 (1H, m, H-12β), 0.97 (3H, d, *J* = 6.6 Hz, H-16), 0.88 (3H, t, *J* = 6.6 Hz, H-10′), 0.82 (3H, s, H-18), 0.79 (3H, s, H-19), 0.67 (3H, s, H-20). ^13^C-NMR (CDCl_3_, 100 MHz) δ: 179.57 (C-15), 173.14 (C-1′), 145.21 (C-8), 112.08 (C-17), 76.06 (C-7), 52.38 (C-9), 48.76 (C-5), 42.06 (C-3), 41.10 (C-14), 39.50 (C-10), 38.79 (C-1), 35.23 (C-12), 34.93 (C-2′), 33.27 (C-18), 33.07 (C-4), 31.86 (C-8′), 30.80 (C-13), 29.47 (C-6′), 29.31 (C-7′), 29.17 (C-5′), 29.07 (C-4′), 28.96 (C-6), 25.24 (C-3′), 22.67 (C-9′), 21.39 (C-19), 20.53 (C-11), 19.96 (C-16), 19.33 (C-2), 14.12 (C-10′), 13.55 (C-20).

*7-O-Lauroyl salvic acid* (**17**) (Yield: 81%, 250 mg); ^1^H-NMR (CDCl_3_, 400 MHz) δ: 5.41 (1H, s, H-7), 5.15 (1H, s, H-17α), 4.71 (1H, s, H-17β), 2.38 (1H, dd, *J* = 15.0; 5.4 Hz, H-14α), 2.29 (2H, t, *J* = 7.4 Hz, H-2′), 2.11 (1H, dd, *J* = 15.0; 8.6 Hz, H-14β), 1.95 (1H, m, H-9), 1.92 (1H, m, H-13), 1.88 (1H, m, H-6α), 1.76 (1H, d, *J* = 12.6 Hz, H-1α), 1.60 (2H, m, H-3′), 1.56 (1H, m, H-6β), 1.52 (2H, m, H-2), 1.48 (1H, s, H-5), 1.44 (1H, m, H-12α), 1.40 (1H, s, H-3α), 1.29 (2H, m, H-11), 1.26-1.29 (14H, m, H-4′ to H-10′), 1.26 (2H, m, H-11′), 1.19 (1H, td, *J* = 13.3; 4.2 Hz, H-3β), 1.06 (1H, dd, *J* = 13.5; 4.3 Hz, H-1β), 1.03 (1H, dd, *J* = 12.7; 4.9 Hz, H-12β), 0.98 (3H, d, *J* = 6.6 Hz, H-16), 0.88 (3H, t, *J* = 6.7 Hz, H-12′), 0.82 (3H, s, H-18), 0.79 (3H, s, H-19), 0.67 (3H, s H-20). ^13^C-NMR (CDCl_3_, 100 MHz) δ: 178.73 (C-15), 173.10 (C-1′), 145.26 (C-8), 112.07 (C-17), 76.08 (C-7), 52.43 (C-9), 48.79 (C-5), 42.09 (C-3), 41.09 (C-14), 39.53 (C-10), 38.83 (C-1), 35.28 (C-12), 34.94 (C-2′), 33.27 (C-18), 33.08 (C-4), 31.92 (C-10′), 30.83 (C-13), 29.66 (C-9′), 29.63 (C-6′), 29.36 (C-8′), 29.34 (C-7′), 29.19 (C-5′), 28.99 (C-4′), 28.99 (C-6), 25.25 (C-3′), 22.69 (C-11′), 21.40 (C-19), 20.57 (C-11), 19.97 (C-16), 19.34 (C-2), 14.12 (C-12′), 13.56 (C-20).

### 3.7. Esterification and Etherification of Salvic Acid: 7-Alkoxy, 15 Ester Derivatives *(**18**–**21**)*

A solution of 200 mg (0.62 mmol) of salvic acid **1** in the appropriate type of anhydrous alcohol (methanol, ethanol, 1-butanol, or 1-propanol; 5 mL) was treated with two drops of 85% H_2_SO_4_ and was heated to reflux for 1 h. The solvent was evaporated under reduced pressure, and the residue was redissolved in CH_2_Cl_2_ (10 mL) and washed with a 5% aqueous solution of NaHCO_3_ (2 × 10 mL) and H_2_O. After drying with anhydrous Na_2_SO_4_, the solvent was evaporated to yield a yellowish oil, which was purified by CC with CH_2_Cl_2_ to yield the respective derivatives. To differentiate the C and H atoms present in the labdanes, the acyl group of the C-7 position was labelled using the prime (′) and (″) double prime symbol. Enumeration was performed in ascending order, starting with 1′ for the C=O carbon and 1″ for the adjacent carbon of alkoxy group.

*Methyl O-methylsalvate* (**18**) (Yield: 72%, 156 mg); the spectral data of **18** were in agreement with those published results [15].

*Ethyl O-ethylsalvate* (**19**) (Yield: 90%, 211 mg); ^1^H-NMR (CDCl_3_, 400 MHz) δ: 4.98 (1H, s, H-17α), 4.67 (1H, s, H-17β), 4.12 (2H, q, *J* = 7.1 Hz, H-1′), 3.85 (1H, t, *J* = 2.7 Hz, H-7), 3.35 (1H, dq, *J* = 9.6; 7.1 Hz, H-1α″), 3.24 (1H, dq, *J* = 9.7; 7.0 Hz, H-1β″), 2.31 (1H, dd, *J* = 14.4; 5.9 Hz, H-14α), 2.09 (1H, dd, *J* = 14.5; 8.2 Hz), 1.94 (1H, m, H-13), 1.93 (1H, m, H-9), 1.88 (1H, dt, *J* = 13.7; 2.3 Hz, H-6α), 1, 71 (1H, d, *J* = 12.5 Hz, H-1α), 1.57 (1H, s, H-5), 1.50 (2H, m, H-2), 1.47 (1H, m, H-6β), 1.44 (1H, m, H-12α), 1.38 (1H, d, *J* = 12.8 Hz, H-3α), 1.25 (3H, t, *J* = 7.1 Hz, H-2″), 1.24 (2H, m, H-11), 1.21 (1H, m, H-3β), 1.15 (3H, t, *J* = 7.0, H-2″), 1.08 (1H, td, *J* = 12.8, 3.9 Hz, H-1β), 0.98 (1H, m, H-12β), 0.94 (3H, d, *J* = 6.7 Hz, H-16), 0.87 (3H, s, H-18), 0.78 (3H, s, H-19), 0.65 (3H, s, H-20). ^13^C-NMR (CDCl_3_, 101 MHz) δ: 173.37 (C-15), 148.06 (C-8), 110.11 (C-17), 80.48 (C-7), 62.21(C-1″), 61.00 (C-1′), 51.37 (C-9), 48.17 (C-5), 42.10 (C-3), 41.70 (C-14), 39.73 (C-10), 38.76 (C-1), 35.26 (C-12), 33.39 (C-18), 33.14 (C-4), 31.14 (C-13), 30.18 (C-6), 21.53 (C-19), 20.40 (C-16), 20.02 (C-11), 19.48 (C-2), 15.36 (C-2″), 14.31 (C-2′), 13.71 (C-20).

*Propyl O-propylsalvate* (**20**) (Yield: 82%, 206 mg); ^1^H-NMR (CDCl_3_, 400 MHz) δ: 4.98 (1H, s, H-17α), 4.67 (1H, s, H-17β), 4.03 (2H, t, *J* = 6.7 Hz, H-1′), 3.82 (1H, s, H-7), 3.18 (3H, m, H-1″), 2.33 (1H, dd, *J* = 14.4; 5.8 Hz, H-14β), 1.94 (1H, m, H-9), 1.90 (1H, m, H-13), 1.71 (1H, d, *J* = 12.6 Hz, H-1α),1.65 (2H, m, H-2″), 1.60 (1H, m, H-6), 1.56 (1H, m, H-5), 1.54 (2H, m, H-2″), 1.50 (1H, m, H-2), 1.38 (1H, d, *J* = 13.1 Hz, H-3α), 1.25 (1H, s, H-11), 1.21 (1H, m, H-3β), 1.08 (1H, dd, *J* = 13.0; 4.1 Hz, H-1β), 0.99 (2H, m, H-12), 0.94 (3H, d, *J* = 6.5 Hz, H-16), 0.93 (3H, t, *J* = 7.5 Hz, H-3′), 0.89 (3H, t, *J* = 7.5 Hz, H-3″), 0.87 (3H, s, H-18), 0.78 (3H, s, H-19), 0.65 (3H, s, H-20). ^13^C-NMR (CDCl_3_, 101 MHz) δ: 173.50 (C-15), 148.05 (C-8), 110.08 (C-17), 80.60 (C-7), 68.60 (C-1″), 65.81 (C-1′), 51.37 (C-9), 48.17 (C-5), 42.10 (C-3), 41.68 (C-14), 39.68 (C-10), 38.78 (C-1), 35.29 (C-12), 33.39 (C-18), 33.11 (C-4), 31.11 (C-13), 30.16 (C-6), 23.02 (C-2″), 22.03 (C-2′), 21.51(C-19), 20.36 (C-16), 20.01 (C-11), 19.46 (C-2), 13.69 (C-20), 10.90 (C-3″) 10.46 (C-3′).

*Butyl O-butylsalvate* (**21**) (Yield: 84%, 228 mg); ^1^H-NMR (CDCl_3_, 400 MHz) δ: 4.97 (1H, s, H-17α), 4.67 (1H, s, H-17β), 4.06 (2H, t, *J* = 6.7 Hz, H-1′), 3.81 (1H, s, H-7), 3.26 (1H, dt, *J* = 9.5; 6.3 Hz, H-1α″), 3.18 (1H, dt, *J* = 9.5; 6.3 Hz, H-1β″), 2.32 (1H, dd, *J* = 14-5; 5.8 Hz, H-14α), 2.08 (1H, dd, *J* = 14.5, 8.3 Hz, H-14β), 1.93 (1H, m, H-9), 1.90 (1H, m, H-13), 1.86 (1H, m, H-6α), 1.71 (1H, d, *J* = 12.5 Hz, H-1α), 1.60 (2H, m, H-2′), 1.56 (1H, m, H-5), 1.51 (2H, m, H-2″), 1.50 (2H, m, H-2), 1.49 (2H, m, H-3″), 1.48 (1H, m, H-6β), 1.46 (1H, m, H-12α), 1.39 (1H, m, H-3α), 1.37 (2H, m, H-3′), 1.28 (2H. m, H-11), 1.21 (1H, td, *J* = 13.2, 4.0 Hz, H-3β), 1.07 (1H, td, *J* = 12.8, 4.0 Hz, H-1β), 0.99 (1H, m, H-12β), 0.95 (3H, m, H-16), 0.93 (3H, t, *J* = 7.2 Hz, H-4′), 0.91 (3H, t, *J* = 7.3 Hz, H-4″), 0.86 (3H, s, H-18), 0.78 (3H, s, H-19), 0.65 (3H, s, H-20). ^13^C-NMR (CDCl_3_, 101 MHz) δ: 173.46 (C-15), 148.09 (C-8), 110.05 (C-17), 80.74 (C-7), 66.86 (C-1″), 64.06 (C-1′), 51.43 (C-9), 48.20 (C-5), 42.13 (C-3), 41.68 (C-14), 39.69 (C-10), 38.81 (C-1), 35.36 (C-12), 33.39 (C-18), 33.12 (C-4), 32.00 (C-2″), 31.12 (C-13), 30.76 (C-2′), 30.19 (C-6), 21.52 (C-19), 20.38 (C-11), 20.38 (C-16), 19.63 (C-3″), 19.48 (C-2), 19.20 (C-3), 14.08 (C-4″), 13.73 (C-4′), 13.70 (C-20).

### 3.8. Purity of the Evaluated Diterpenes

The purity of each diterpene **1**–**21**, (Scheme 1) was estimated by thin-layer chromatography using different solvent systems. The compounds were also submitted for ^1^H- and ^13^C-NMR spectral data analysis, which indicated a purity of 95–98% for each compound.

### 3.9. Antimicrobial Assays

The MIC (the lowest concentration at which the compound inhibited microbial growth) and MIA (the lowest amount at which the compound inhibited microbial growth) of the hemisynthetic derivatives of salvic acid were determined in triplicate using the microdilution broth method in 96-well microplates in liquid media and diffusion techniques for the agar overlay method in solid media. Standard strains of the following microorganisms were obtained from the American Type Culture Collection: Gram-negative bacterium *E. coli* (АТCC 25922) and Gram-positive bacteria *S. aureus* (ATCC 6538) and *B. cereus* (ATCC 13061). Samples of the compounds were dissolved in CH_3_OH (methanol) at 2 mg·mL^−1^ and were subsequently diluted in methanol to achieve concentrations ranging from 2000 to 50 μg·mL^−1^. Methanol was used as a negative control. The inoculum was adjusted for each organism to yield a cell concentration of 1.5 × 10^8^ colony forming units (CFU)/mL.

#### 3.9.1. Solid Media Bioassays

To a Petri dish containing tryptic soy agar medium (TSA) on 1.5% agar (20 mL) (BD, Becton Drive, Franklin Lakes, NJ, USA), 3 mL of tryptic soy broth medium (TSB) (BD, Becton Drive, Franklin Lakes, NJ, USA) was added, along with 0.75% agar melted at 55 °C, which was previously inoculated with 100 µL of a bacterial culture grown for 18 h (overnight) and diluted to 0.5 Mc Farland, equivalent to 1.5 × 10^8^ CFU/mL. Once solidified, a 5 μL aliquot of the methanolic dilution of each compound at various concentrations was deposited on the bacterial lawn, along with 5 μL of methanol as a solvent control. After incubation at 37 °C for 18 h, the diameter of the inhibition zone was examined. The antibacterial activity was determined by the presence or absence of a clear halo in the bacterial lawn. The MIA corresponded to dilutions with lowest compound concentrations, producing a clear halo. Penicillin, ciprofloxacin, kanamycin, tetracycline, and chloramphenicol were used as positive controls.

#### 3.9.2. Liquid Media Bioassays

Assays were performed in 96-well plates containing 188 μL of Mueller-Hinton broth (MHB), 2 μL of a bacterial culture grown overnight and diluted to 0.5 Mc Farland, and 10 μL of each compound. In addition, the absorbance of methanolic dilutions of each compound in 190 μL of MHB and 10 μL of the target compound was determined as a control. The other controls included a growth control (GC): 198 μL of MHB + 2 μL of inoculum, inhibition control (IC): 188 μL of MHB + 2 μL of inoculum + 10 μL of chloramphenicol at 0.05 μg/μL, solvent control (SolC): 180 μL of MHB + 2 μL of inoculum + 10 μL of methanol, and sterility control (SteC): 200 μL of MHB. The plates were incubated overnight at 37 °C, and the optical density (OD) was measured at 600 nm using a spectrophotometric detector for microplates (ELISA reader). All tests were performed in triplicate.

### 3.10. Docking Study

A palmitoyloleylphophatidylglycerol bilayer composed of 128 units of POPG of dimensions 40 × 40 × 40 Å^3^ was built with the aid of VMD software [44]. The geometric-optimized molecular structures of all compound derivatives were calculated using the density functional theory method (DFT) based in Becke three-parameter, Lee-Yang-Parr (B3LYP) functional, and 6-31G basis set, and their partial charges were calculated using CHarges from ELectrostatic Potentials using a Grid based method (CHelpG) option at a standard 6-31G level. The resulting structures were then docked into the bilayer with AutoDock4 [45].

### 3.11. Estimated Lipophilicity Values

Lipophilicity values have been estimated with the aid of the XLOGP3 program. The XLOGP3 method estimates logP values for structures related with reference compounds for which experimental log *p* values are available [46]. The additive model implemented in XLOGP3 uses a total of 87 atom/group types and two correction factors as descriptors. It is calibrated on a training set of 8199 organic compounds with reliable logP data through a multivariate linear regression analysis. It is comparable with other methods, with average errors in the range of ±0.24–0.51 units.

## 4. Conclusions

Salvic acid **1** (7α-hydroxy-8(17)-*ent-*labden-15-oic acid), a labdane with two HBD groups (hydroxyl in C-7 and carboxylic acid in C-15), was used to perform a structure-activity study to evaluate the effect of lipophilicity and HBD groups on the antibacterial activity of labdanes. The experimental results showed a selective in vitro inhibitory activity against Gram-positive bacteria *S. aureus* and *B. cereus*. In structural terms, the modification of the HBD group of the C-15 position changing the carboxylic acid for the hydroxyl group (compound **2**), and blocking out the two HBD groups for alkoxy in C-7 and the esters group in C-15 (compounds **18**–**21**) eliminates the antibacterial activity. The suppression of one HBD group by modification of the carboxylic acid by a methylester group in C-15 (compound **3**) and acylation of C7-OH (compounds **4**–**15**) exhibited an increase of activity. In the 7-*O*-acylated derivatives, increase in the alkyl chain length in the acyl group correlated with an increase in activity of up to five carbons and decreased and disappeared as the chain length increased to nine carbons. Increase in the chain length is in agreement with the lipophilicity increase, which shows that the activity and lipophilicity present a linear correlation up to an optimal length and not ad infinitum. These results provide a reference for medicinal scientists attempting to optimize the natural antibacterial compounds with a labdane skeleton during the drug development stage.

## Figures and Tables

**Table 1:** Minimum inhibitory concentration (MIC), minimum inhibitory amount (MIA), and lipophilicity of salvic acid and its derivatives.

Compound	*S. aureus*		*B. cereus*		*E. coli*		logP ^3^
Salvic acid (**1**)	50.00 ^1^	20.00 ^2^	50.00 ^1^	22.50 ^2^	>100.00 ^1^	>10.00 ^2^	4.90
**2**	>100.00 ^1^	>25.00 ^2^	>100.00 ^1^	>25.00 ^2^	>100.00 ^1^	>10.00 ^2^	5.08
**3**	25.00 ^1^	3.00 ^2^	50.00 ^1^	2.00 ^2^	>100.00 ^1^	>10.00 ^2^	5.23
**4**	50.00 ^1^	4.50 ^2^	25.00 ^1^	4.00 ^2^	>100.00 ^1^	>10.00 ^2^	5.47
**5**	25.00 ^1^	2.50 ^2^	12.50 ^1^	1.50 ^2^	>100.00 ^1^	>10.00 ^2^	5.94
**6**	6.25 ^1^	1.50 ^2^	3.13 ^1^	1.25 ^2^	>100.00 ^1^	>10.00 ^2^	6.30
**7**	6.25 ^1^	1.25 ^2^	6.25 ^1^	1.00 ^2^	>100.00 ^1^	>10.00 ^2^	6.52
**8**	3.13 ^1^	0.75 ^2^	3.13 ^1^	0.50 ^2^	>100.00 ^1^	>10.00 ^2^	6.84
**9**	3.13 ^1^	0.50 ^2^	3.13 ^1^	0.30 ^2^	>100.00 ^1^	>10.00 ^2^	6.74
**10**	3.13 ^1^	0.75 ^2^	3.13 ^1^	0.40 ^2^	>100.00 ^1^	>10.00 ^2^	6.92
**11**	3.13 ^1^	0.35 ^2^	3.13 ^1^	0.25 ^2^	>100.00 ^1^	>10.00 ^2^	7.38
**12**	3.13 ^1^	1.00 ^2^	3.13 ^1^	0.75 ^2^	>100.00 ^1^	>10.00 ^2^	7.55
**13**	6.25 ^1^	1.25 ^2^	6.25 ^1^	1.25 ^2^	>100.00 ^1^	>10.00 ^2^	7.13
**14**	3.13 ^1^	0.40 ^2^	3.13 ^1^	0.45 ^2^	>100.00 ^1^	>10.00 ^2^	8.47
**15**	12.50 ^1^	3.00 ^2^	6.25 ^1^	2.50 ^2^	>100.00 ^1^	>10.00 ^2^	9.01
**16**	>100.00 ^1^	>10.00 ^2^	25.00 ^1^	>10.00 ^2^	>100.00 ^1^	>10.00 ^2^	9.55
**17**	>100.00 ^1^	>10.00 ^2^	>100.00 ^1^	>10.00 ^2^	>100.00 ^1^	>10.00 ^2^	10.63
**18**	>100.00 ^1^	>10.00 ^2^	>100.00 ^1^	>10.00 ^2^	>100.00 ^1^	>10.00 ^2^	5.76
**19**	>100.00 ^1^	>10.00 ^2^	>100.00 ^1^	>10.00 ^2^	>100.00 ^1^	>10.00 ^2^	6.50
**20**	>100.00 ^1^	>10.00 ^2^	>100.00 ^1^	>10.00 ^2^	>100.00 ^1^	>10.00 ^2^	7.55
**21**	>100.00 ^1^	>10.00 ^2^	>100.00 ^1^	>10.00 ^2^	>100.00 ^1^	>10.00 ^2^	8.26
Penicillin	0.03 ^1^	1.23 ^2^	2.50 ^1^	5.00 ^2^	n/t	n/t	-
Ciprofloxacin	0.25 ^1^	2.50 ^2^	0.13 ^1^	2.50 ^2^	n/t	n/t	-
Kanamycin	1.00 ^1^	2.50 ^2^	1.00 ^1^	5.00 ^2^	n/t	n/t	-
Tetracycline	1.00 ^1^	1.23 ^2^	5.00 ^1^	5.00 ^2^	n/t	n/t	-
Chloramphenicol	2.00 ^1^	1.50 ^2^	0.25 ^1^	0.25 ^2^	n/t	n/t	-
Methanol	i ^1^	i ^2^	i ^1^	i ^2^	i ^1^	i ^2^	-

^1^ MIC in liquid media (μg/mL); ^2^ MIA in solid media (μg); ^3^ XlogP3, n/t: not tested i: inactive.

## Supplementary Materials

Supplementary materials are available online.

## Abbreviations

The following abbreviations are used in this manuscript:

ATCC	American type culture collection.
B3LYP	Becke three-parameter, Lee-Yang-Parr
CC	Column chromatography
CFU	Colony forming units
CHelpG	CHarges from ELectrostatic Potentials using a Grid
CLSI	Clinical Laboratory Standards
DFT	Density functional theory
GC	growth control
HBA	Hydrogen bond acceptor
HBD	Hydrogen bond donor
IC	inhibition control
logP_ow_	Octanol–water partition coefficient
MHB	Mueller-Hinton broth
MIA	Minimum inhibitory amount
MIC	Minimum inhibitory concentration
NMR	Nuclear magnetic resonance
OD	optical density
POPG	1-palmitoyl-2-oleoyl-sn-glycero-3-[phospho-rac-(1-glycerol)]
SAR	Structure-activity relationships
SolC	Solvent control
SteC	Sterility control
TSA	Tryptic soy agar media
TSB	Tryptic soy broth media
