# Supplementary material for: Enhanced Antibacterial Activity of Ent-Labdane Derivatives of Salvic Acid (7α-Hydroxy-8(17)-ent-Labden-15-Oic Acid): Effect of Lipophilicity and the Hydrogen Bonding Role in Bacterial Membrane Interaction
Source: Molecules. 2017 Jun 23;22(7):1039. doi: 10.3390/molecules22071039 (PMC6152121; doi:10.3390/molecules22071039)
Supplement: Supplementary file 1 [file molecules-22-01039-s001.pdf]

Article

Supplementary Materials: Enhanced Antibacterial Activity of *ent*-labdane Derivatives of Salvic Acid (7α-hydroxy-8(17)-*ent*-Labden-15-Oic Acid): Effect of Lipophilicity and the Hydrogen Bonding Role in Bacterial Membrane Interaction

Javier Echeverría [1,*], Alejandro Urzúa [2], Loreto Sanhueza [3] and Marcela Wilkens [4]

1. General Information

NMR spectra were obtained on a Bruker DPX 400 spectrometer (400 MHz for ^{1}H and 100 MHz for ^{13}C). Samples were dissolved in $CDCl_3$, and the spectra were calibrated using TMS signals. The chemical shifts are given in ppm. The carbon atoms in the alkyl chains of the acyloxy groups were numbered by labelling the carbon atom of the carbonyl group as number one and subsequently increasing towards the methyl terminus of the acyl chain.

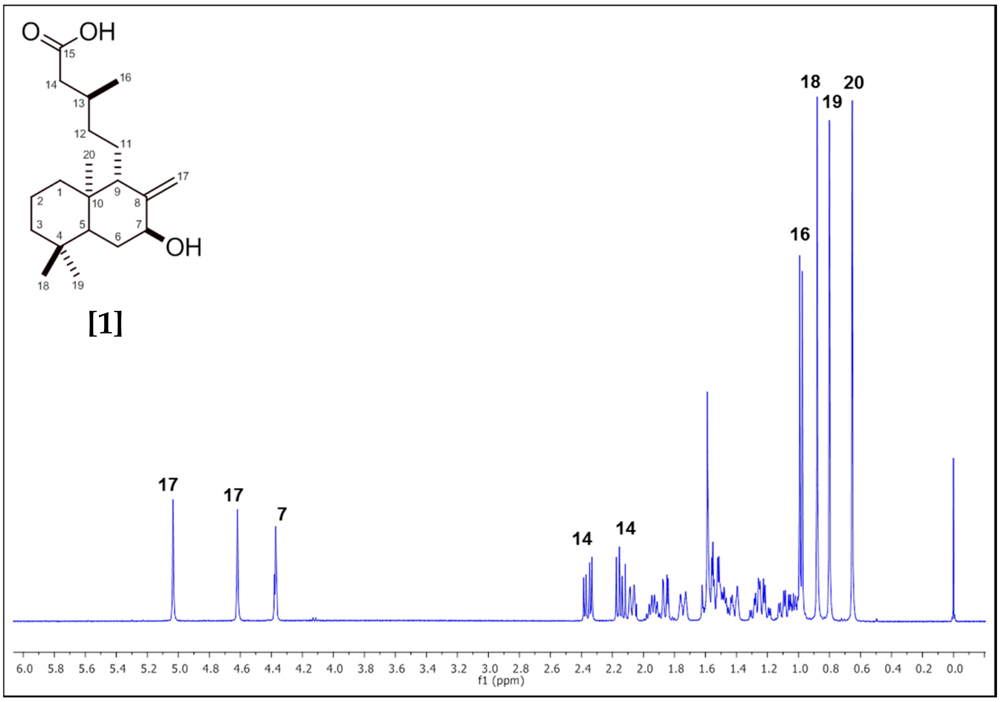

Figure. S1 ^{1}H-NMR of salvic acid (**1**)

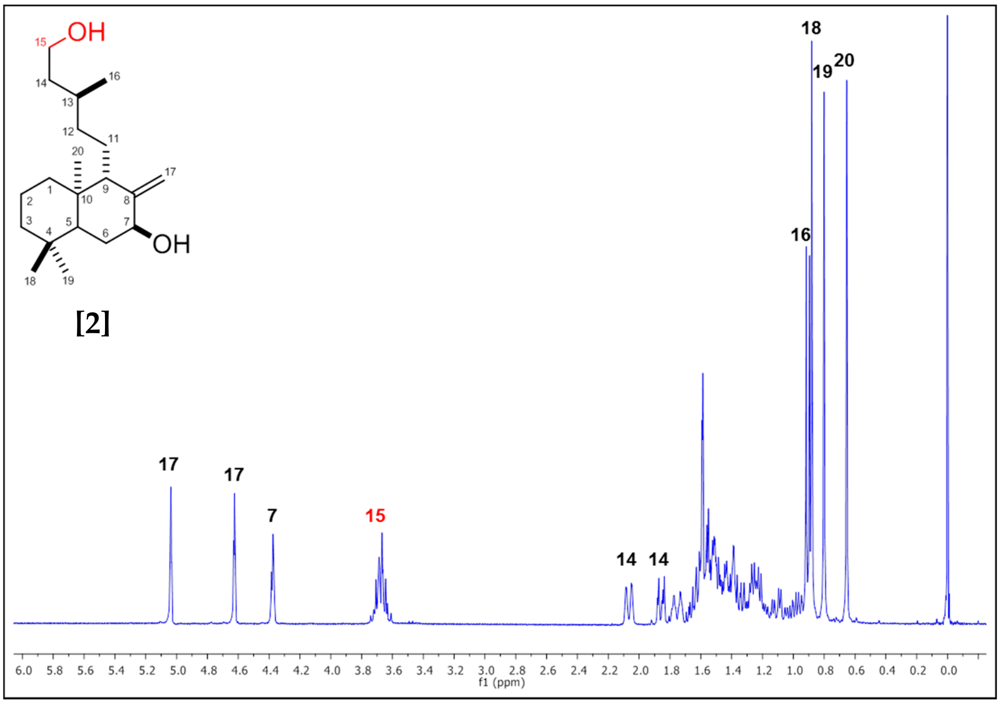

Figure. S2 ^{1}H-NMR of 7β,15-dihydroxy-*ent*-lab-8(17)-ene (**2**)

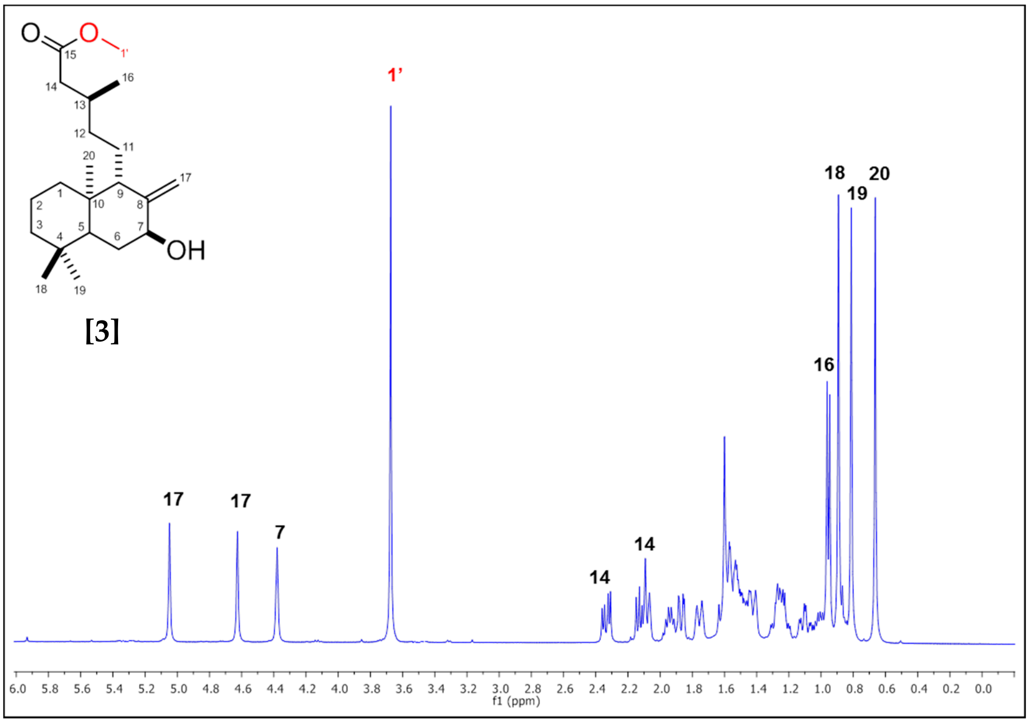

Figure. S3 ^{1}H-NMR of Methylsalvate (**3**)

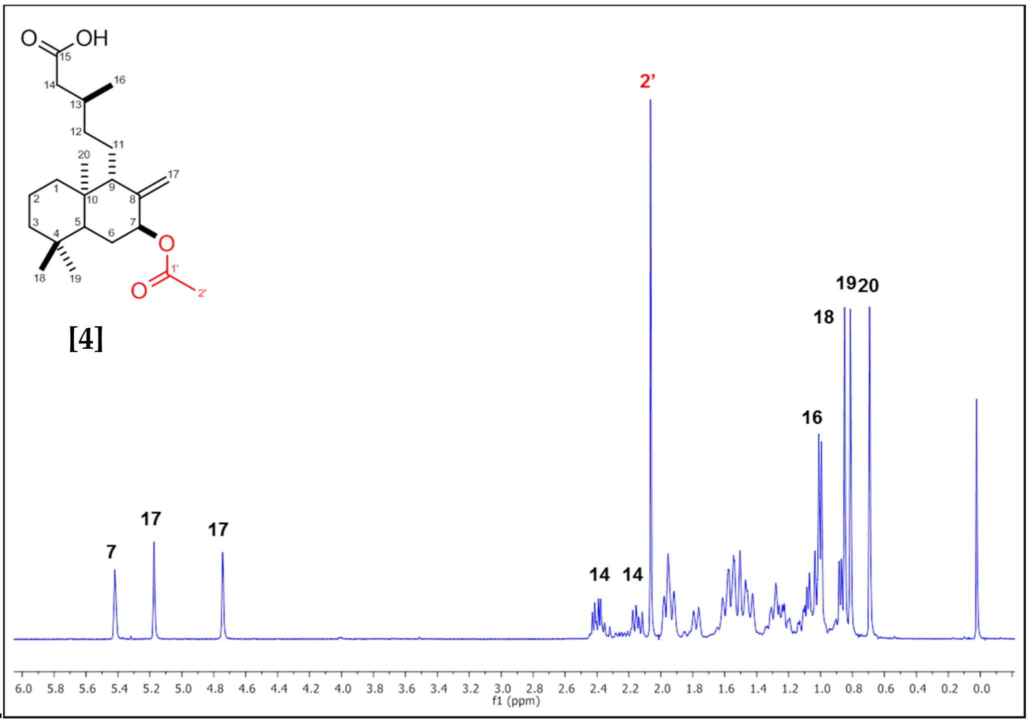

Figure.S4 ^{1}H-NMR of 7-*O*-acetyl salvic acid (**4**)

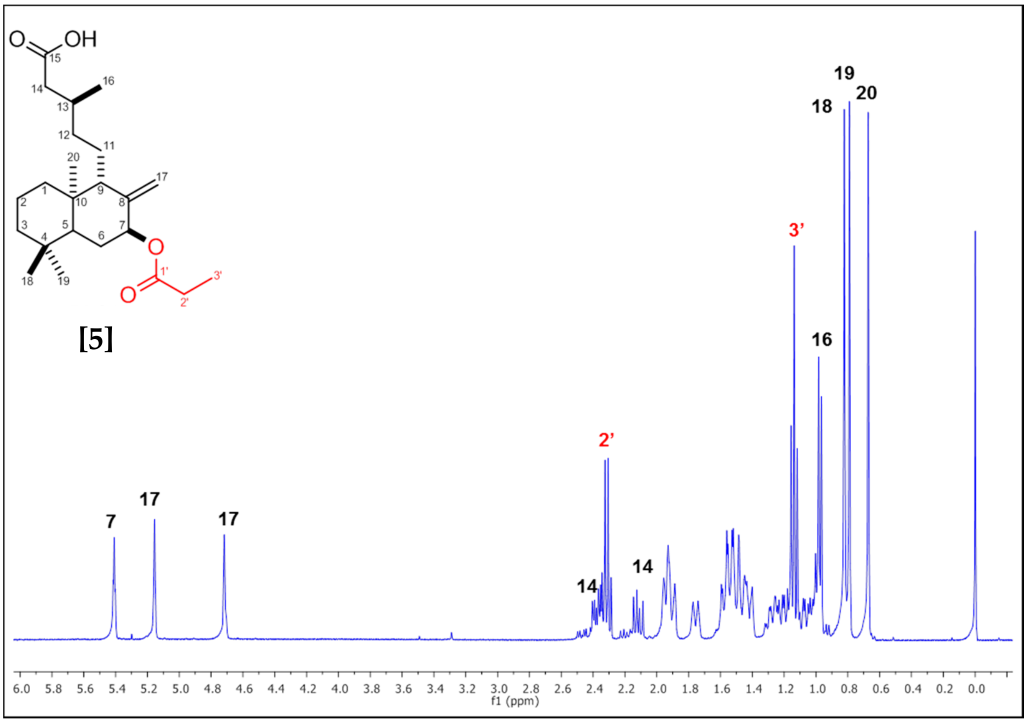

Figure. S5 ^{1}H-NMR of 7-*O*-propionyl salvic acid (**5**)

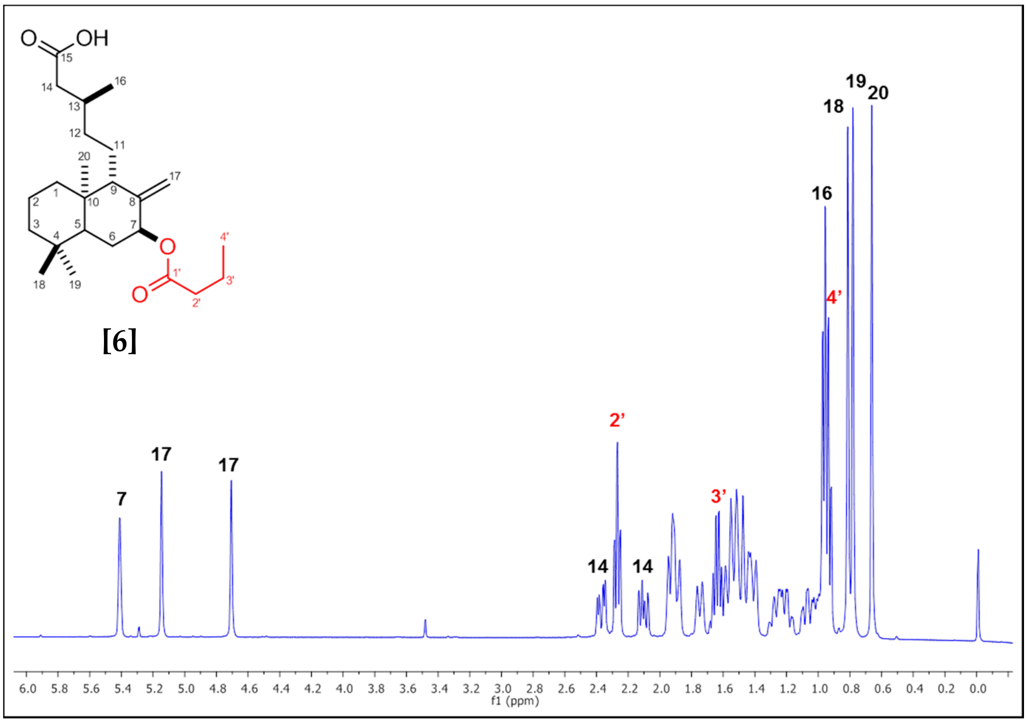

Figure. S6 ^{1}H-NMR of 7-*O*-butyryl salvic acid (**6**)

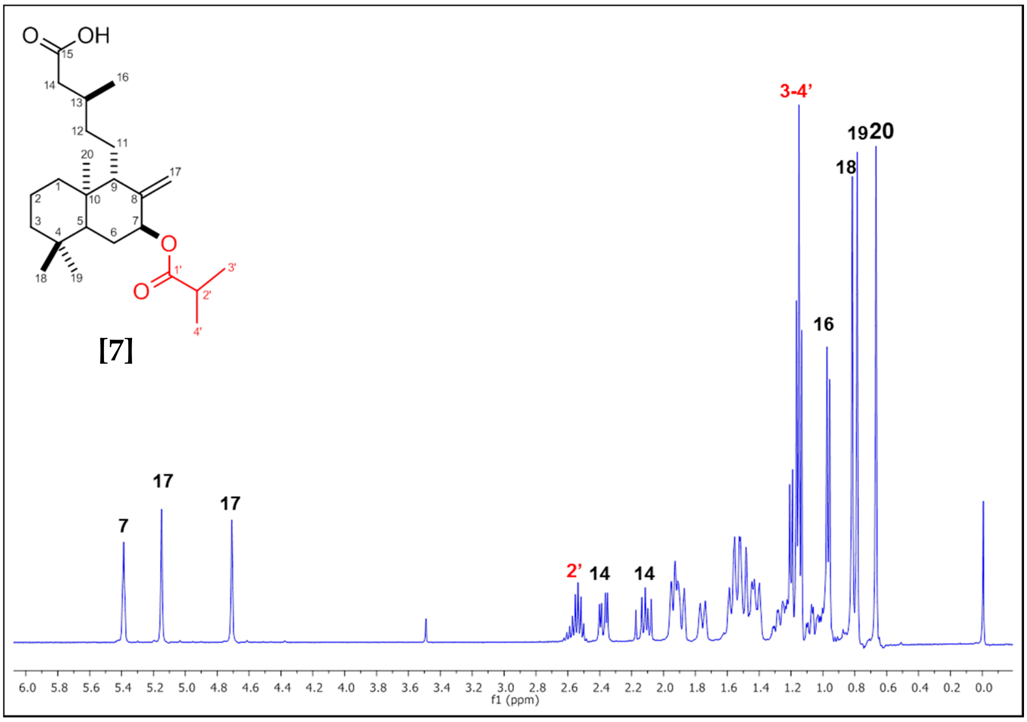

Figure. S7 ^{1}H-NMR of 7-*O*-isobutyryl salvic acid (**7**)

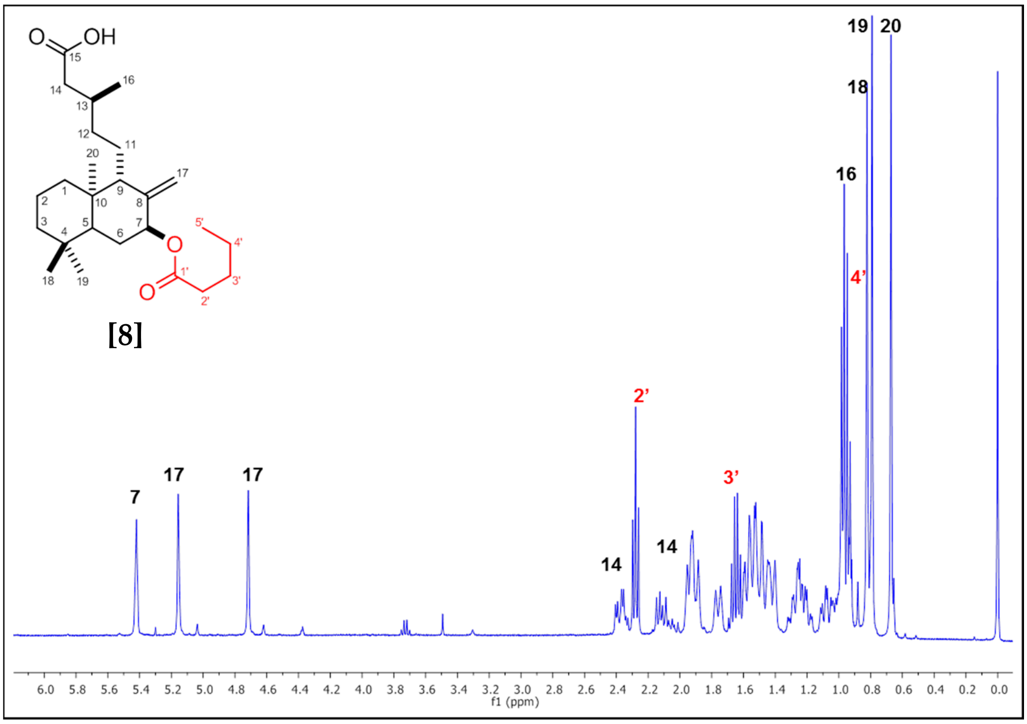

Figure. S7 ^{1}H-NMR of 7-*O*-valeroyl salvic acid (**8**)

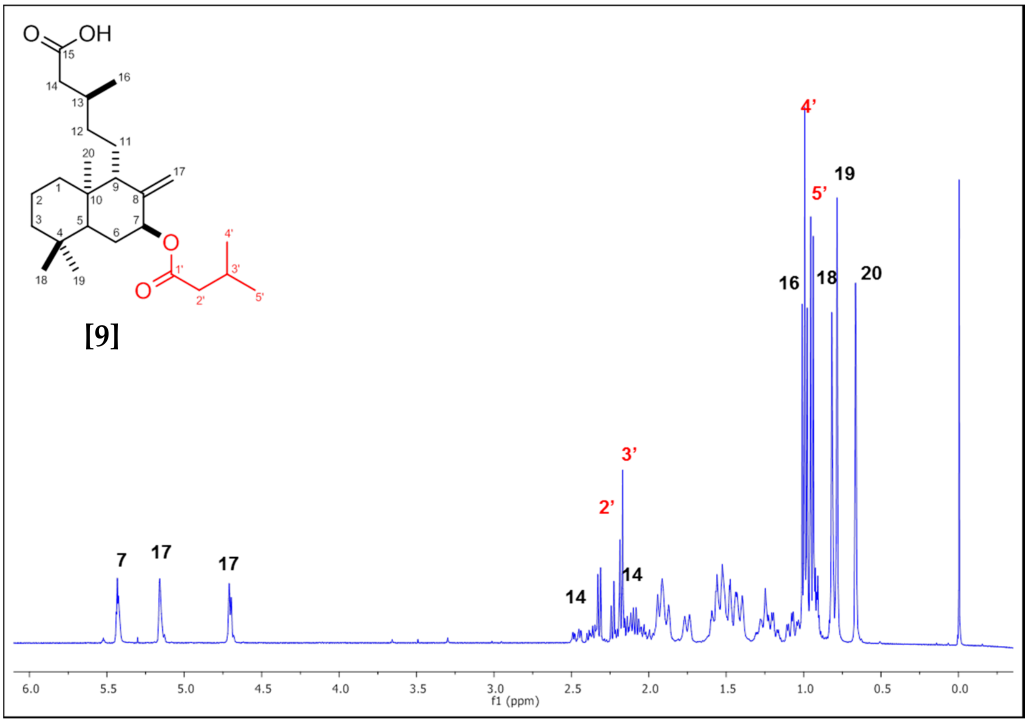

Figure. S9 ^{1}H-NMR of 7-*O*-isovaleroyl salvic acid (**9**)

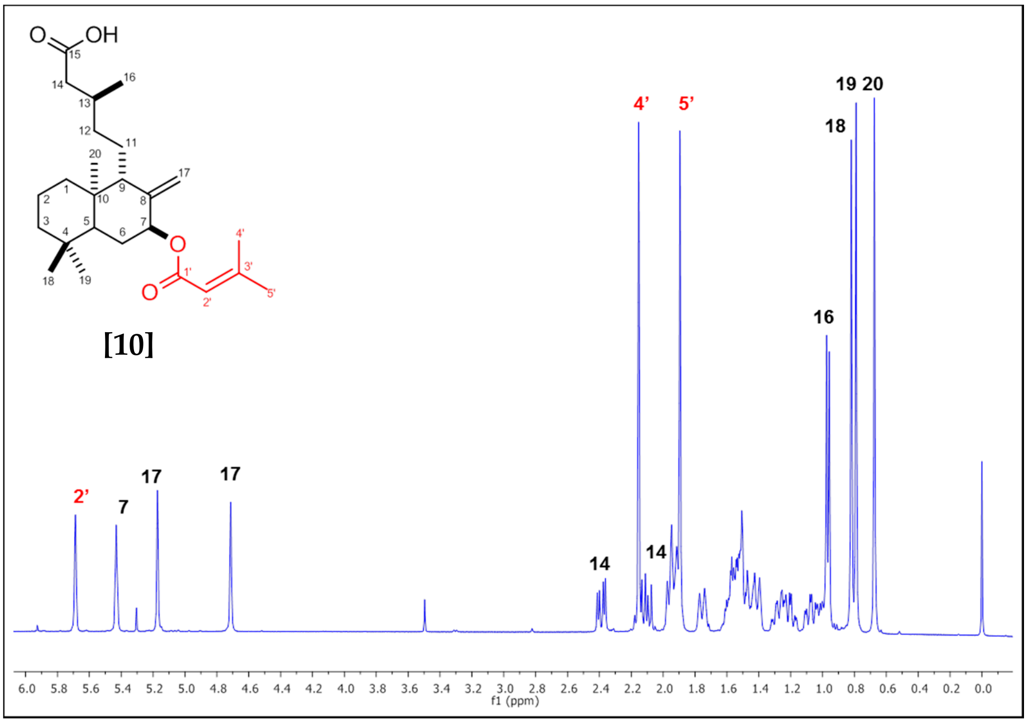

Figure. S10 ^{1}H-NMR of 7-*O*-senecioyl salvic acid (**10**)

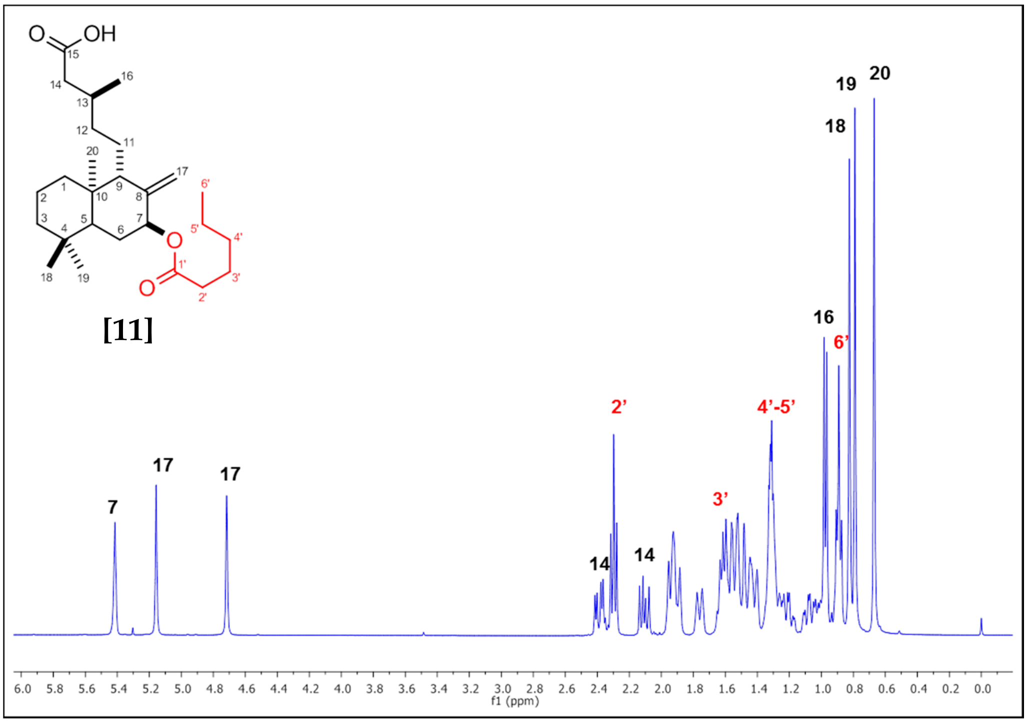

Figure. S11 ^{1}H-NMR of 7-*O*-cyclohexanoyl salvic acid (**11**)

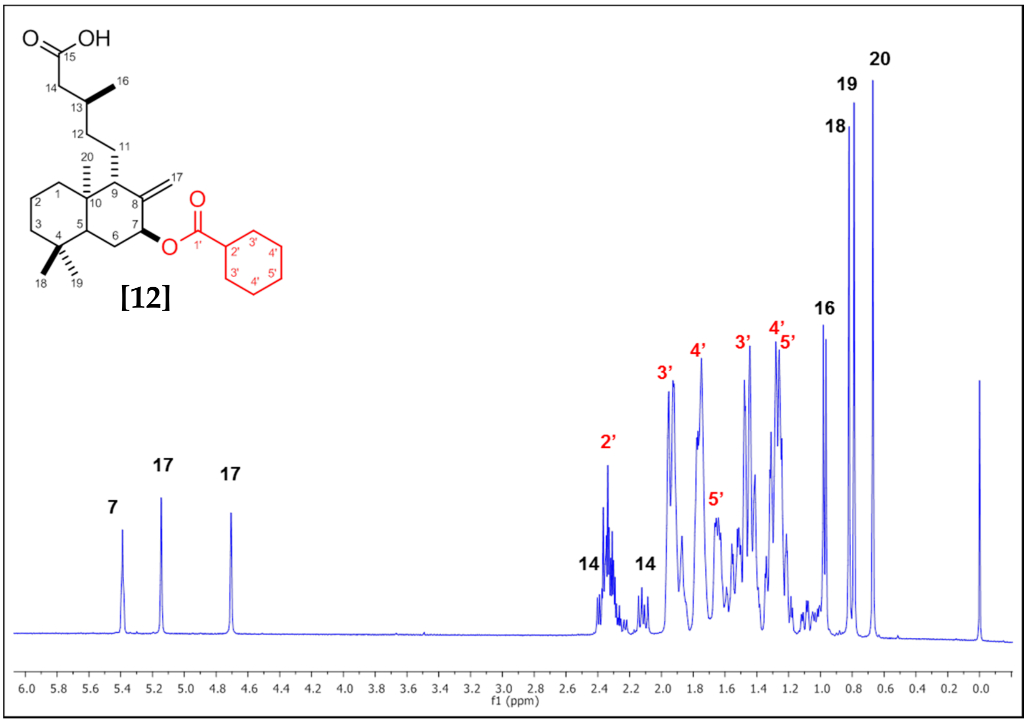

Figure. S12 ^{1}H-NMR of 7-*O*-cyclohexanoyl salvic acid (**12**)

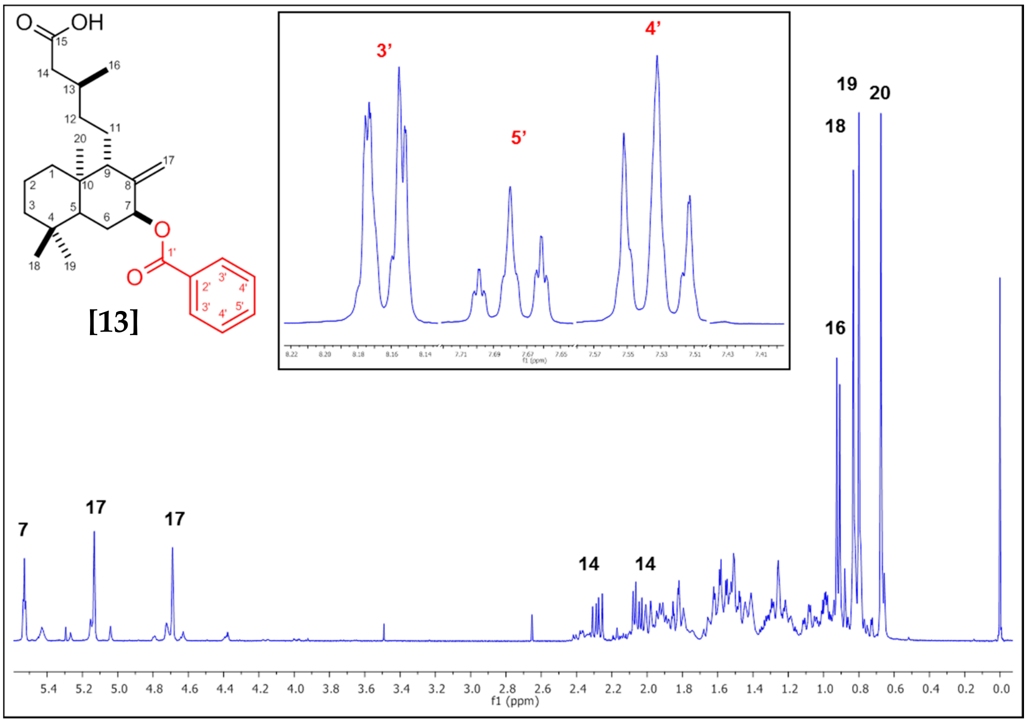

Figure. S13 ^{1}H-NMR of 7-*O*-benzoyl salvic acid (**13**)

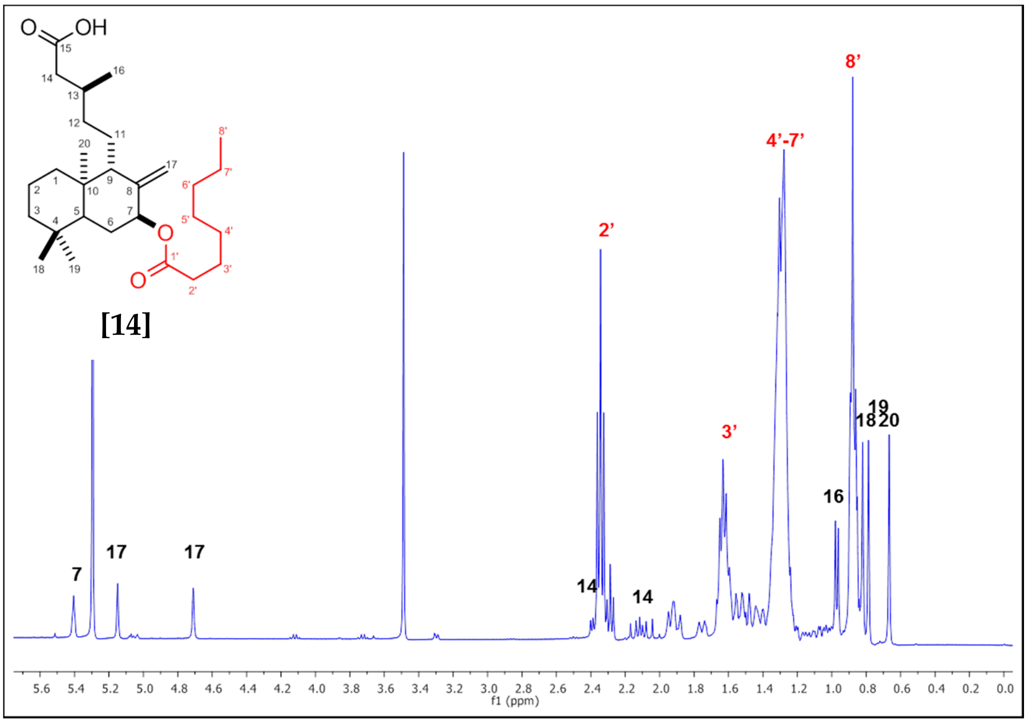

Figure. S14 ^{1}H-NMR of 7-*O*-octanoyl salvic acid (**14**)

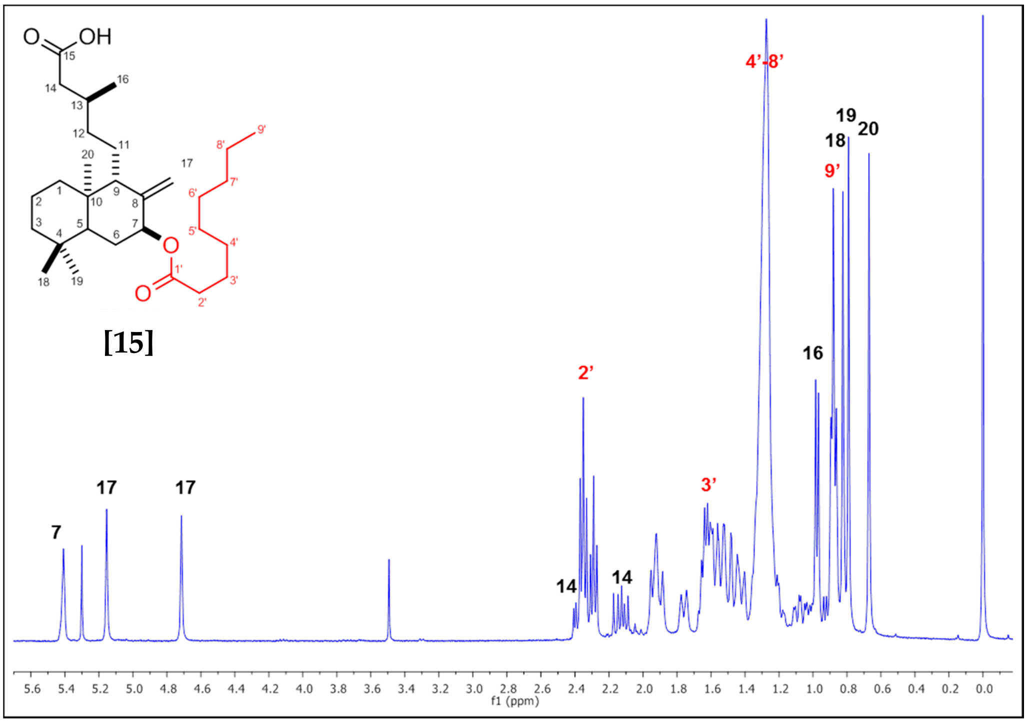

Figure. S15 ^{1}H-NMR of 7-*O*-pelargonoyl salvic acid (**15**)

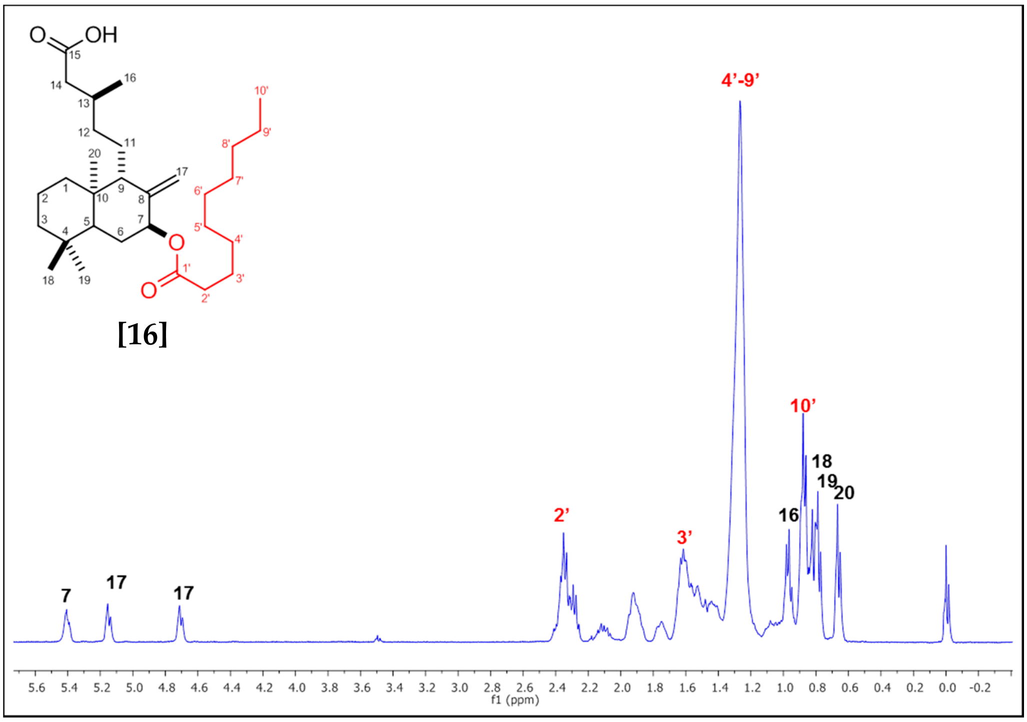

Figure. S16 ^{1}H-NMR of 7-*O*-decanoyl salvic acid (**16**)

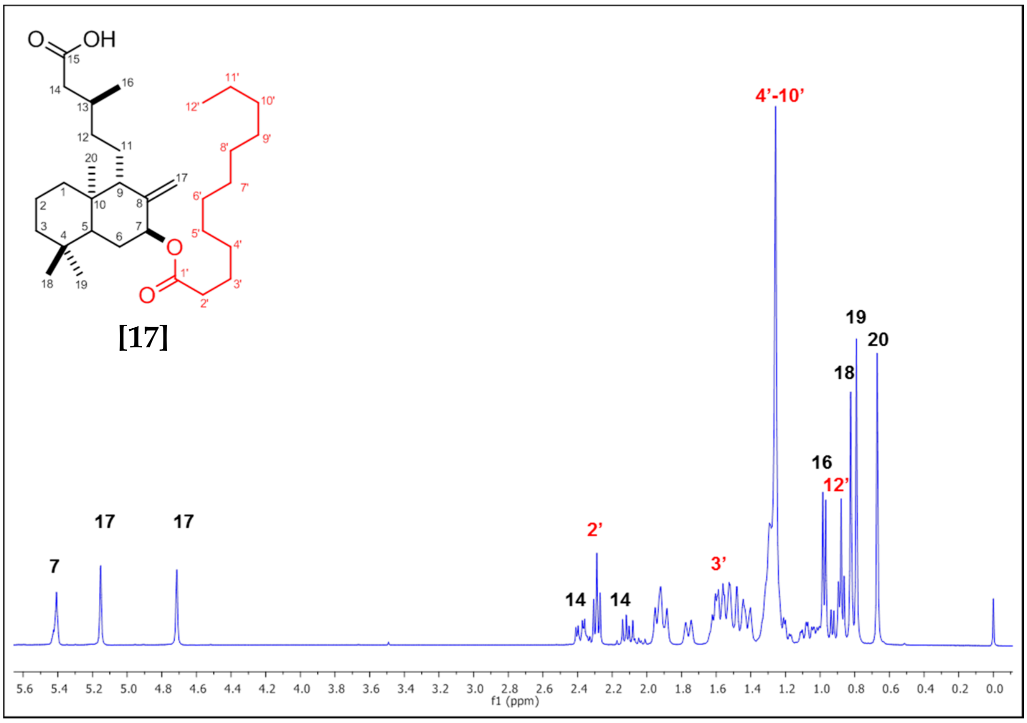

Figure. S17 ^{1}H-NMR of 7-*O*-lauroyl salvic acid (**17**)

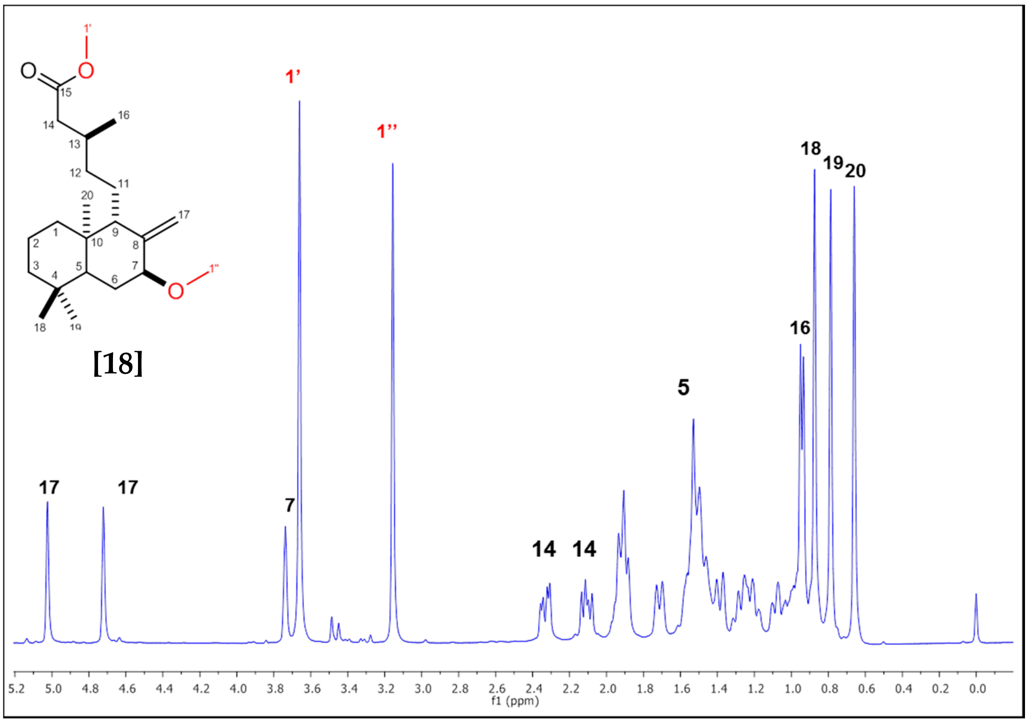

Figure. S18 ^{1}H-NMR of Methyl *O*-methylsalvate

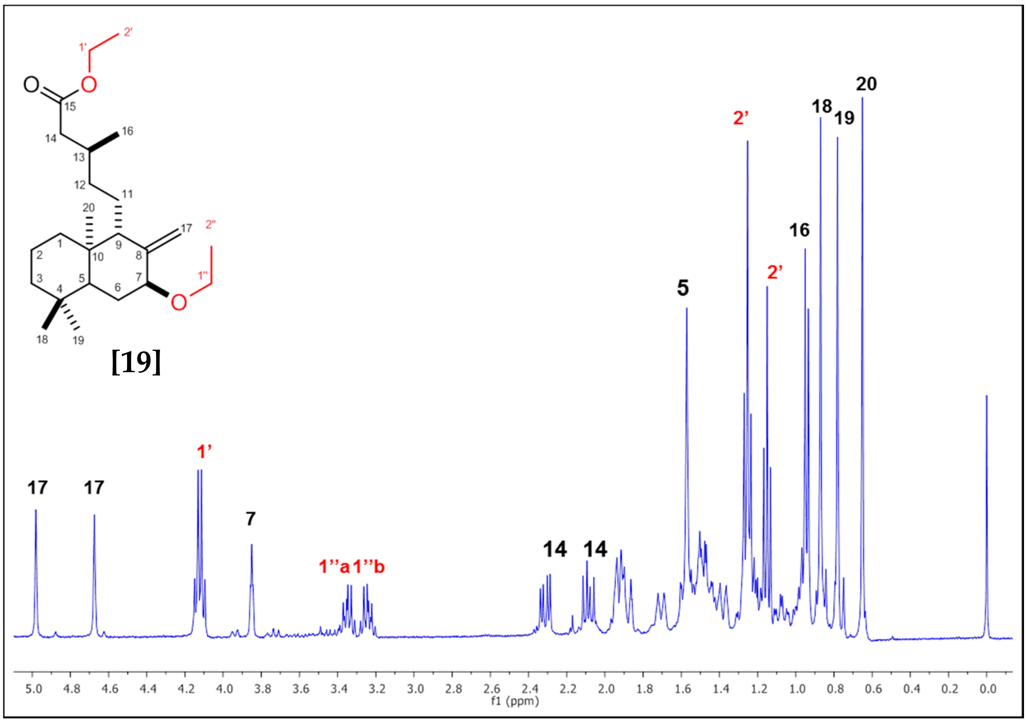

Figure. S19 ^{1}H-NMR of Ethyl *O*-ethylsalvate (**19**)

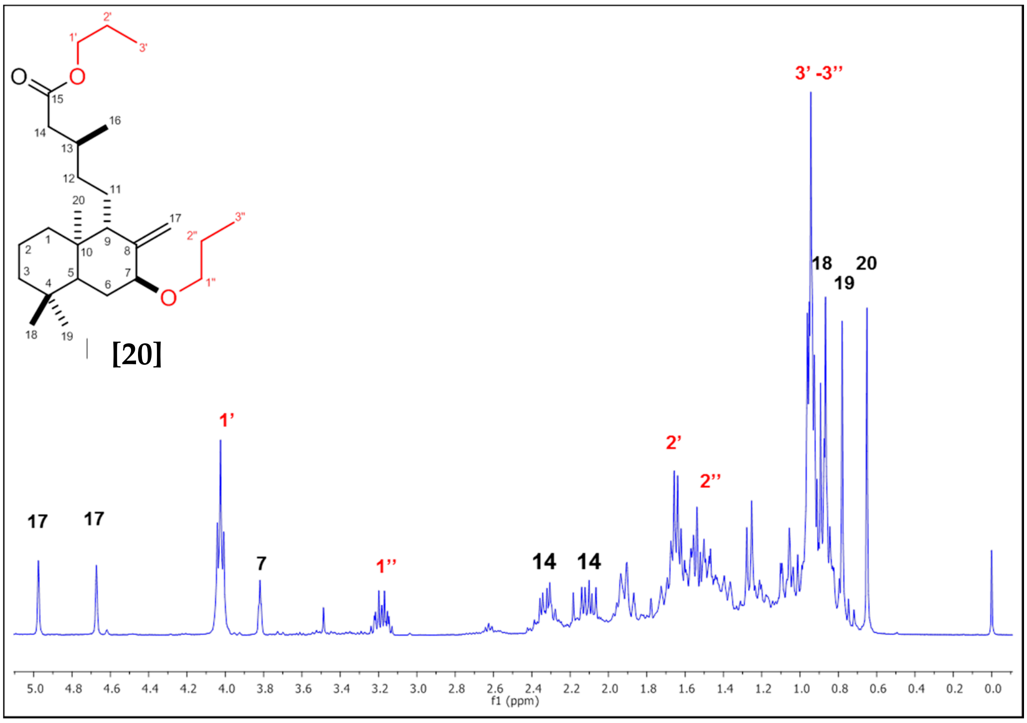

Figure. S20 ^{1}H-NMR of Propyl *O*-propylsalvate (**20**)

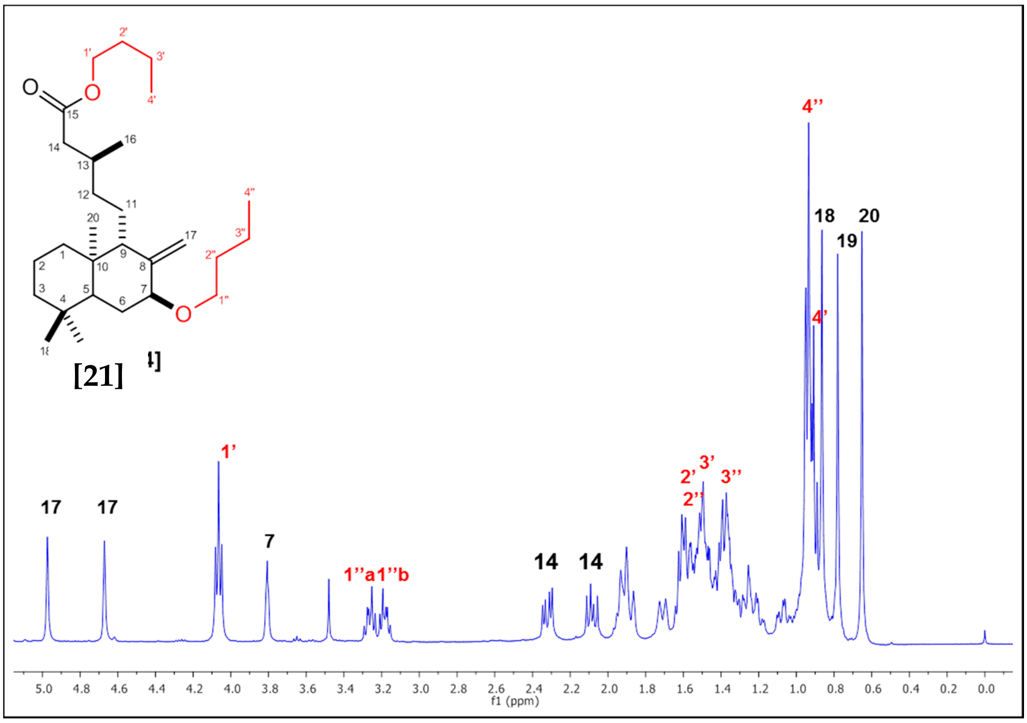

Figure. S21 ^{1}H-NMR of Butyl *O*-butylsalvate (**21**)
